# FcRn Rescues Recombinant Factor VIII Fc Fusion Protein from a VWF Independent FVIII Clearance Pathway in Mouse Hepatocytes

**DOI:** 10.1371/journal.pone.0124930

**Published:** 2015-04-23

**Authors:** Arjan van der Flier, Zhan Liu, Siyuan Tan, Kai Chen, Douglas Drager, Tongyao Liu, Susannah Patarroyo-White, Haiyan Jiang, David R. Light

**Affiliations:** Hematology Research, Biogen, Cambridge, Massachussets, United States of America; Emory University School of Medicine, UNITED STATES

## Abstract

We recently developed a longer lasting recombinant factor VIII-Fc fusion protein, rFVIIIFc, to extend the half-life of replacement FVIII for the treatment of people with hemophilia A. In order to elucidate the biological mechanism for the elongated half-life of rFVIIIFc at a cellular level we delineated the roles of VWF and the tissue-specific expression of the neonatal Fc receptor (FcRn) in the biodistribution, clearance and cycling of rFVIIIFc. We find the tissue biodistribution is similar for rFVIIIFc and rFVIII and that liver is the major clearance organ for both molecules. VWF reduces the clearance and the initial liver uptake of rFVIIIFc. Pharmacokinetic studies in FcRn chimeric mice show that FcRn expressed in somatic cells (hepatocytes or liver sinusoidal endothelial cells) mediates the decreased clearance of rFVIIIFc, but FcRn in hematopoietic cells (Kupffer cells) does not affect clearance. Immunohistochemical studies show that when rFVIII or rFVIIIFc is in dynamic equilibrium binding with VWF, they mostly co localize with VWF in Kupffer cells and macrophages, confirming a major role for liver macrophages in the internalization and clearance of the VWF-FVIII complex. In the absence of VWF a clear difference in cellular localization of VWF-free rFVIII and rFVIIIFc is observed and neither molecule is detected in Kupffer cells. Instead, rFVIII is observed in hepatocytes, indicating that free rFVIII is cleared by hepatocytes, while rFVIIIFc is observed as a diffuse liver sinusoidal staining, suggesting recycling of free-rFVIIIFc out of hepatocytes. These studies reveal two parallel linked clearance pathways, with a dominant pathway in which both rFVIIIFc and rFVIII complexed with VWF are cleared mainly by Kupffer cells without FcRn cycling. In contrast, the free fraction of rFVIII or rFVIIIFc unbound by VWF enters hepatocytes, where FcRn reduces the degradation and clearance of rFVIIIFc relative to rFVIII by cycling rFVIIIFc back to the liver sinusoid and into circulation, enabling the elongated half-life of rFVIIIFc.

## Introduction

Hemophilia A is an X-linked bleeding disorder caused by the deficiency of coagulation Factor VIII and is currently treated by intravenous injection of replacement factor VIII, either as on-demand or prophylaxis therapy [1]. Recombinant factor VIII Fc fusion protein (rFVIIIFc), a long-acting factor VIII composed of a single B domain-deleted (BDD) human FVIII covalently attached to the Fc domain of human IgG1 [2], was designed to increase the circulating half-life of FVIII by enabling entry of rFVIIIFc into the IgG recycling pathway following endocytosis. The Fc region of rFVIIIFc binds to the neonatal Fc receptor (FcRn), and studies in FcRn knock-out mice confirmed a role for FcRn in prolonging the half-life of rFVIIIFc [2]. Additionally, phase 1/2a and 3 (A-LONG) studies demonstrated an ~1.5-fold extended half-life of rFVIIIFc relative to rFVIII in patients with hemophilia A, as well as efficacy and safety for the prevention and control of bleeding episodes [3,4].

The neonatal Fc receptor (FcRn) is a heterodimer composed of an MHC class I-like molecule (encoded by the *Fcgrt* gene) and β2-microglobulin and is part of a natural pathway that rescues plasma IgG and albumin following endocytosis by diverting them from lysosomal degradation and cycling them back into circulation [5–9]. FcRn plays a role in a number of biological processes including immunity [10] and maternal-fetal transfer of IgG [11] and is expressed in many tissues, including somatic cells (epithelial, endothelial, and hepatocytes) and most hematopoietic cells, except T-cells or NKT-cells. Both endothelial and hematopoietic FcRn-expressing cells protect circulating IgG from degradation, as shown in studies with FcRn bone marrow chimeric mice [12–14] or conditional knockout mice where FcRn is deleted in both endothelial and hematopoietic cells [15]. Since uptake is dictated by the expression of protein-specific clearance receptors, it is unknown if cells that contribute to the decreased clearance of IgG by FcRn-mediated rescue are the same or different from those cells involved in the uptake and cycling of rFVIIIFc or recombinant factor IX Fc fusion protein (rFIXFc) [16].

FVIII is synthesized and secreted by both liver sinusoidal endothelial and extrahepatic endothelial cells [17,18] which maintain normal FVIII plasma levels of 0.5 to 1 nM (100 to 250 ng/mL) in humans [19]. Most FVIII circulates bound to the large multimeric glycoprotein VWF [20]. Plasma VWF levels are in 30 to 50-fold molar excess over endogenous FVIII when quantified as total VWF monomers (~50 nM based on VWF level of 8 to 12 μg/mL) [21]. Most circulating plasma VWF originates from endothelial cells which can constitutively secrete VWF and by a regulated secretory pathway from Weibel-Palade bodies, in addition VWF is also secreted following platelet activation [22]. The dynamic association between VWF and FVIII stabilizes FVIII and protects it from proteolytic degradation [23,24] and receptor mediated clearance [25], increasing both FVIII plasma levels and circulating half-life [26]. Von Willebrand disease patients who do not express VWF or who express the type 2N variant with impaired FVIII binding show decreased FVIII plasma levels of <10% of normal [27,28].

The clearance receptors and cell types involved in the clearance of FVIII and VWF are not well established. The scavenger receptors implicated in the clearance of FVIII, VWF or the complex of FVIII and VWF, as identified by receptor binding, receptor knockout mice, or linkage studies of the human genome include low-density lipoprotein receptor-related protein-1 (LRP1), low-density lipoprotein receptor (LDLR), asialoglycoprotein receptor (ASGPR), macrophage mannose receptor type 1 (MMR/CD206), heparan sulfate proteoglycans, sialic acid binding IgG-like lectin 5 (Siglec5), scavenger receptor class A member 5 (SCARA5), stabilin-2 (STAB2) and C-type lectin domain family 4 member M (CLEC4M).[29–36]. Immunohistochemistry studies demonstrate both murine liver Kupffer cells and macrophages are involved in the uptake of VWF and FVIII [37,38].

Our objective was to determine the cellular distribution of rFVIIIFc, relative to that of rFVIII, in the organs, tissues and cells responsible for their clearance. We wanted to identify the cell types in which the interaction between the Fc domain and FcRn contributes to the decreased clearance of rFVIIIFc. We investigated how VWF affects the clearance and biodistribution of both rFVIIIFc and rFVIII in mice deficient in both FVIII and VWF (FVIII/VWF-DKO), in order to determine whether the complex of rFVIIIFc and VWF is recycled by FcRn.

## Materials and Methods

### Reagents

rFVIIIFc was produced as described previously [2]. BDD-rFVIII and rFVIIIFc mutants, rFVIIIFc-IHH (I253A, H310A, H435A, defective in FcRn binding) [39] and rFVIIIFc-N297A (defective in FcRγ binding) [40] were produced similarly in HEK-293 cells. rFIXFc was prepared as described previously [16]. Antibodies are listed in S1 Table. RNeasy RNA isolation kit (Qiagen), Taqman reverse transcription reagents, Universal PCR master mix and Taqman RT-PCR primers (S2 Table) were all from Invitrogen.

### Mice

FVIII deficient mice (B6;129S-*F8* ^*tm1Kaz*^/J, FVIII-KO) [41] were obtained from Dr. Haig H. Kazazian (University of Pennsylvania, Philadelphia, PA). VWF deficient mice (B6.129S2-*VWF* ^*tm1Wgr*^/J, VWF-KO) [42] were obtained from Dr. Denisa Wagner (Harvard Medical School, Boston, MA). FVIII and VWF double knockout mice (FVIII/VWF-DKO) were generated by crossing FVIII-KO and VWF-KO mice to double homozygosity. FcRn bone marrow chimeric and control mice were created from FcRn knockout (B6.129X1-*Fcgrt* ^*tm1Dcr*^/DcrJ, FcRn-KO) [43] and wild-type isogenic C57BL/6 mice: CD45.1 isogenic, B6.SJL-Ptprca Pep3b/BoyJ or CD90.1 isogenic, B6.PL-*Thy1* ^*a*^/CyJ (Jackson Laboratory).

### Ethics statement

All animal studies were approved by the Institutional Animal Care and Use Committee (IACUC) of Biogen Idec (Permit Number: 01–10) and performed in accordance with the Guide to the Care and Use of Laboratory Animals to minimize animal suffering [44].

### Biodistribution studies with iodinated rFVIII or rFVIIIFc

rFVIII and rFVIIIFc were iodinated with ^125^I (~ one iodine label per molecule) using the Bolton-Hunter protocol [45], under conditions that allow retention of 80% of FVIII activity as verified by the one-stage aPTT assay (final specific activity of cold iodinated ^127^I-rFVIIIFc was 7330 IU/mg compared to control FVIIIFc specific activity of 9243 IU/mg). Coagulation activity pre- and post-iodination was measured using a one-stage activated partial thromboplastin time (aPTT) assay described previously [4]. For organ scintillation counting, male FVIII-KO or FVIII/VWF-DKO mice (8–10 weeks) were provided 20 mM KI in drinking water to minimize free ^125^I-iodine uptake into the thyroid for 72 hours prior to dosing with 14 μCi/mouse of ^125^I-rFVIII or ^125^I-rFVIIIFc (~80μg/kg). For each time-point two mice were euthanized by CO_2_ inhalation and perfused for 5 minute with PBS before organ dissection and weighing. The amount of radioactivity in each sample was quantitated using a gamma-counter. For quantitative whole body autoradiography (QWBA), single mouse per time-point were administered one intravenous dose of 6.5 μCi ^125^I-rFVIIIFc (34 μg/kg), euthanized by CO_2_ inhalation, snap frozen and embedded in 2% carboxymethyl cellulose matrix. Representative (40 μm) sections from 4 to 5 per animal were examined to encompass all organs and structures of interest. Sections were imaged using the Storm 860 image acquisition system (GE-Healthcare Life Sciences). Quantification relative to calibration standards was performed using MCID image analysis software (GE-Healthcare Life Sciences).

### Generation of FcRn bone marrow chimeric mice

Marrow was isolated from donor mice and 5x 10^6^ bone marrow cells were transplanted intravenously via the tail vein into 7 to 10 week, lethally irradiated recipient mice (split dose of 2x 750 rad). Each cohort encompassed 4 groups of 10 mice each, wild-type (WT→WT) and FcRn deficient (KO→KO) controls and chimeras expressing FcRn in hematopoietic cells (WT→KO) or expressing FcRn in somatic cells (KO→WT). Irradiation resistant Kupffer cells were depleted by dosing 10 ml/kg clodrosomes (Encapsula NanoSciences) at 5 weeks post-transplant and subsequently replenished by donor derived marrow cells [46]. PK studies were performed 10 weeks post-transplant and were reproduced in two independent cohorts (except rFVIII control). Cohort chimerism was determined by flow cytometry of blood leucocytes using isogenic markers CD45.1 and CD45.2 or thymocyte markers CD90.1 and CD90.2. Kupffer cell chimerism was confirmed on 2 cohorts (5 mice per group) by immunohistochemistry of OCT cryosections from acetone post-fixed liver sections using CD45.1/CD45.2 and F4/80 triple staining [46] and quantified using Visiopharm software (Hoersholm, Denmark).

### Pharmacokinetic studies in mice

The PK of rFVIII and rFVIIIFc were determined after a single intravenous dose of 200 or 250 IU/kg, a human FVIII dose frequently used in murine PK studies [47–49]. Citrated blood was collected, under isoflurane anesthesia, by retro-orbital puncture from 3 or 4 mice for each time point (3 time points per mouse). FVIII activity in FVIII-KO and FVIII/VWF-DKO plasma was measured using the Biophen FVIII:C chromogenic activity assay from Hyphen BioMed as described previously [2]. In plasma samples containing endogenous murine FVIII (i.e. FcRn-chimeric mice), human FVIII was first captured by a human FVIII-specific mAb (GMA8016, Green Mountain Antibodies) followed by the FVIII chromogenic activity assay [2]. In PK studies of human IgG1κ (Protos Immunoresearch), 5 mg/kg was dosed, blood was collected daily by tail-tip biopsy, and IgG was detected by ELISA using goat-anti-human #31125 capture and goat-anti-human-HRP #31416 detection pAbs (Pierce). Human rFIXFc was dosed at 250 IU/kg and detected in isolated plasma using a modified human FIX ELISA (AHIX-5041 capture mAb to human FIX, (Haematologic Technologies Inc) and HRP-conjugated goat-anti-human FIX CL20040APHP detection pAb (CedarLane). Murine VWF and IgG1 were quantified by sandwich ELISA (human VWF-EIA, Affinity Biologicals; mouse IgG1 #88-50410-22, eBioscience). PK parameters were estimated by sparse sampling and noncompartmental modeling using Phoenix WinNonlin 6.2.1 (Pharsight, Certara). An unpaired two-tailed t-test was performed in order to determine a statistical significant difference between the plasma FVIII activity levels at each time point using Prism 6.0c Software (GraphPad).

### Immunohistochemistry of rFVIII, rFVIIIFc and VWF in mice

Mice were dosed with equimolar amounts of rFVIII (396 μg/kg) and rFVIIIFc or rFVIIIFc mutants (484 μg/kg). A 5-fold lower molar dose results in an undetectable immunohistochemical signal compared to background under a variety of fixation and perfusion methods tested. Animals were euthanized at 5, 15 and 30 minute or 4 and 5 hour by CO_2_ inhalation and liver slices were snap frozen on dry-ice in OCT (TissueTek, Sakura-Finetek). Cryosections were air-dried, post-fixed with 4% paraformaldehyde in PBS for 10 minute. After PBS washes, the sections were permeabilized with 0.3% Triton X-100 in PBS for 10 minute, washed with PBS and blocked with Tris buffered saline, 0.02% Tween-20, pH 7.5 containing 1% BSA and 5% normal goat serum. All antibody incubations were in blocking buffer, followed by three 5 minute washes with Tris buffered saline, 0.02% Tween-20, pH7.5. Primary antibody dilutions and sources are listed in S1 Table and include: rat-anti-mouse F4/80 (BM8, or CI:A3-1), CD68 (FA-11), CD31 (ER-MP12; or clone 390), rabbit-anti-mouse VWF (Ab6994), anti-human FVIII mAb mix (GMA8004, GMA8009, GMA8018, GMA8019) and incubations were done at 4°C overnight. Secondary antibodies include Alexa-488, -594 or -647 conjugated goat anti-mouse IgG_2a_, goat anti-rat, rabbit or human IgG (Invitrogen), which were incubated for 1 hour at room temperature. Matching image panels of sections embedded in Vectashield mounting media with DAPI (VectorLaboratories) were rendered on a Zeiss LSM710 confocal microscope using ZEN software, 5 μm image stacks merged and final image panels generated (Volocity, Perkin Elmer). For presentation consistency pseudocolors for red and green channels were occasionally switched depending on the secondary antibody fluorophore used (Alexa-488 or Alexa-594). Controls include staining and imaging performed in parallel with non-dosed animals and comparisons to sections stained using antibody incubations lacking one of the primary antibodies (see S4–S8 Figs). Matching acquisition and image processing settings were used for all control supplementary images.

### Primary liver cell isolation

Primary liver cells were isolated as described [50] from livers perfused with Gay Balanced Salt Solution containing 100 U/ml Collagenase IV (CLS-4, Worthington). Hepatocytes were sedimented by centrifugation (2 minutes, 50 x g) and the supernatant containing non-parenchymal cells separated by density centrifugation: liver sinusoidal endothelial cells (LSEC) and Kupffer cells enriched at the 8.2% and 17.6% Optiprep interphases (Sigma-Aldrich). For qPCR analysis, LSEC (CD146 or CD31) and Kupffer cells (anti-F4/80) were further selected using biotin conjugated antibodies and MACS beads (Miltenyi-Biotech).

## Results

### Differential contribution of FcRn-dependent recycling and VWF to the half-life extension of rFVIIIFc

**Fig 1:**
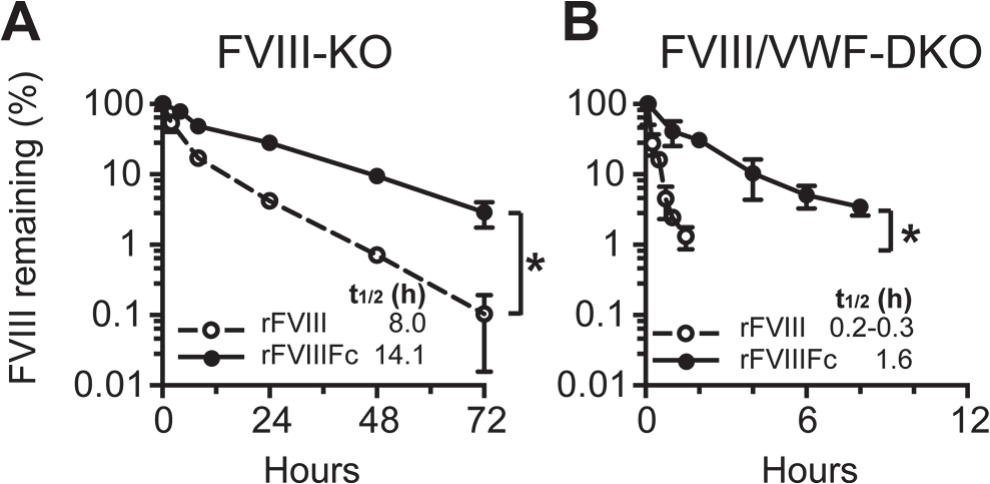
Circulating VWF levels decrease the clearance of rFVIII and rFVIIIFc. Plasma activity of rFVIII and rFVIIIFc versus time profile is shown for (A) FVIII-KO and (B) FVIII/VWF-DKO mice (note the time scale difference). Mice are dosed with 250 IU/kg rFVIII or rFVIIIFc and the FVIII activity remaining in plasma determined by the chromogenic activity assay (3–4 samples per time point, mean ± SD). Significance between plasma levels for individual time points on the PK curves is determined by an unpaired 2-tailed student t-test. A significant difference is indicated between PK curves with one or more significant time point differences (p<0.05).

Previously, we reported that the reduced clearance and prolonged circulating half-life of rFVIIIFc compared to rFVIII depends on expression of FcRn in mice [2]. It is well established that the endogenous level of functional VWF directly controls the level of FVIII in circulation in humans [26,28] as well as mice. We found that the 2-fold difference in the half-life of rFVIII in FVIII-KO mice (mixed C57BL/6/129Sv) versus wild-type C57BL/6 mice (7.6 hour versus 4.3 hour, respectively) [2] correlated with a 2-fold greater plasma VWF level in the FVIII-KO mice relative to the C57BL/6 mice (data not shown). Therefore, we addressed the contribution of endogenous VWF to the clearance of rFVIIIFc relative to rFVIII by measuring the pharmacokinetics of both molecules in FVIII-KO and FVIII/VWF-DKO mice (Fig 1). In FVIII-KO mice, the half-life of rFVIIIFc is 14.1 hours, representing a 1.8-fold increase over rFVIII (8 hours) (Fig 1A). In contrast, the half-life times of rFVIIIFc and rFVIII in FVIII/VWF-DKO mice are 1.6 hour and 20 minute respectively, representing a 5-fold difference in half-life in the absence of VWF (Fig 1B). Indeed, the relatively longer half-life time extension of rFVIIIFc in the absence of VWF indicates that the clearance of both rFVIII and rFVIIIFc bound in the VWF-FVIII complex is the dominant clearance pathway and is dictated by the half-life of VWF itself, which is approximately 13 hours in mice [51]. Recently, similar quantitative effects of VWF in FVIII-KO mice on the clearance of either rFVIII or a glycopegylated rFVIII and their respective mutants defective for binding VWF have been reported [49].

### Biodistribution of rFVIIIFc into the liver is increased in the absence of VWF

**Fig 2:**
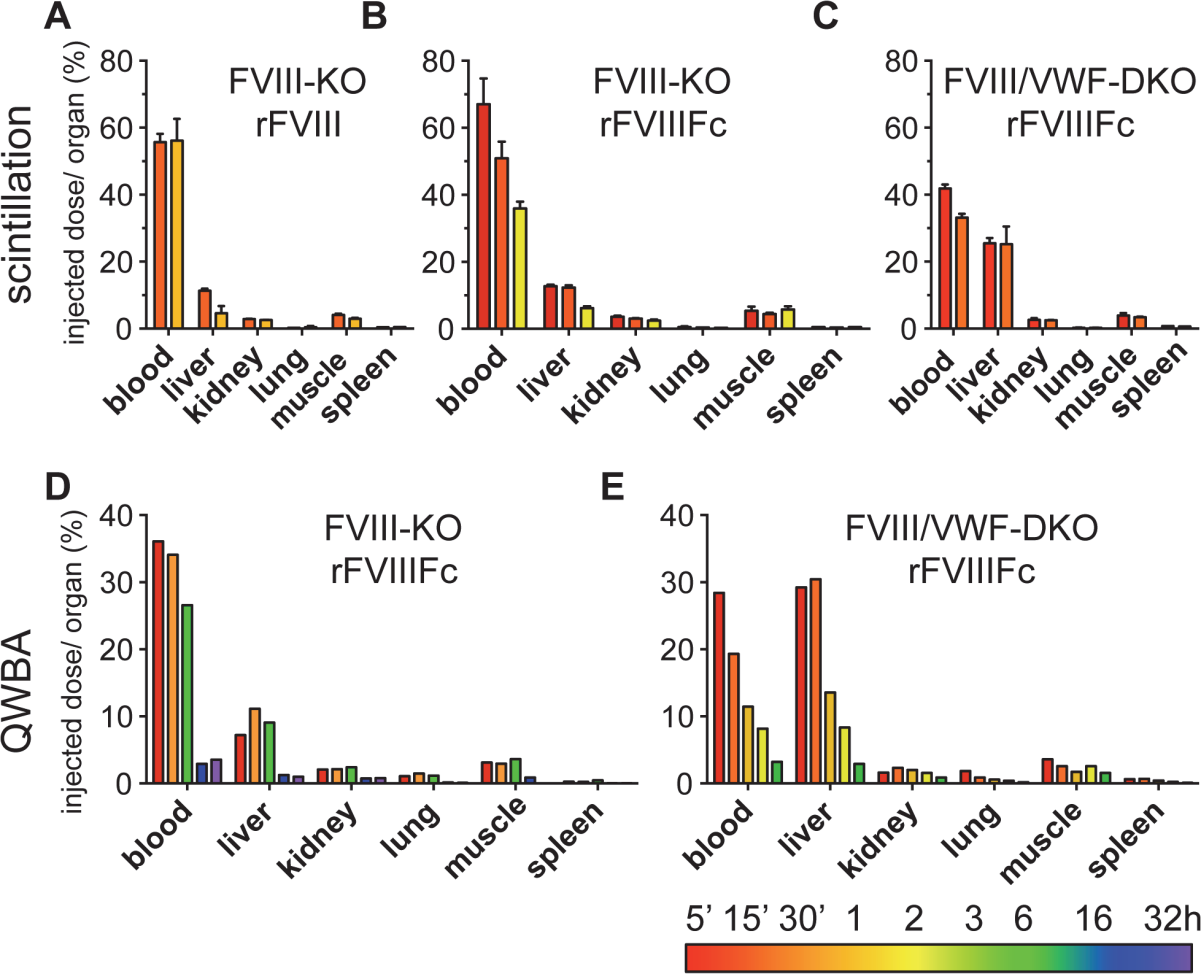
Biodistribution studies demonstrate the liver is the major clearance organ for both rFVIII and rFVIIIFc in mice. (A-C) Quantitation of the radioactivity remaining in perfused organs for ^125^I-rFVIII and ^125^I-rFVIIIFc. (A) rFVIII in FVIII-KO mice (15 and 60 minute) and (B) rFVIIIFc in FVIII-KO mice (5, 15 and 120 minute) show similar biodistributions. (C) rFVIIIFc in FVIII/VWF-DKO mice at early time points (5 and 15 minute) chosen to match the rapid clearance of rFVIIIFc in these mice. (D, E) Biodistribution of ^125^I-rFVIIIFc by QWBA. (D) FVIII-KO mice (5, 30 minute, 3, 16, 32 hour) and (E) FVIII/VWF-DKO (5, 15 minute, 1, 2, 6 hour). Liver is the major clearance organ for both rFVIII and rFVIIIFc and in the absence of endogenous VWF there is a 3-fold increased signal in the liver of rFVIIIFc (C and E). FVIII proteins are labeled using iodination conditions optimized to preserve 80% cofactor activity (Panels A-C; n = 2).

To determine whether VWF affects the tissue biodistribution of rFVIIIFc and rFVIII, the recoveries of radiolabeled rFVIIIFc and rFVIII were quantified in FVIII-KO and FVIII/VWF-DKO mice. The biodistribution of ^125^I-rFVIIIFc and ^125^I-rFVIII was compared in FVIII-KO mice by gamma scintillation counting of blood and weighed samples of perfused organs. In the presence of VWF (in FVIII-KO mice), the biodistribution profile of rFVIIIFc and rFVIII is comparable, with 60 to 70% radioactivity recovery in blood immediately after dosing, 10% recovery in the liver, and lower amounts in the kidneys and spleen (Fig 2A and 2B). In a second study, quantitative whole body analysis (QWBA) following a single dose of ^125^I-rFVIIIFc in FVIII-KO mice confirmed this organ biodistribution profile. Despite the similar organ distribution between the two methods, the overall recovery of rFVIIIFc at the 5 minute time point is much lower by QWBA (50%) than by scintillation counting (90%) as shown in Fig 2B and 2D and S3 and S5 Tables. The major discrepancy between the methods is due to the low recovery in blood by QWBA (36%) compared to scintillation counting (67%). The recovery in blood by scintillation counting is consistent with recoveries observed with FVIII activity assays and ELISA in pharmacokinetic studies, suggesting that the absolute quantitation in blood by QWBA is less accurate.

In agreement with organ scintillation counting, QWBA shows that the liver is the major clearance organ at early time points, accounting for 10% of the injected dose (Fig 2D). In contrast, the recovery of ^125^I-rFVIIIFc in livers of FVIII/VWF-DKO mice increased to 30% by both gamma scintillation counting of perfused organs (Fig 2C) or by QWBA (Fig 2E). The lower ^125^I-rFVIIIFc levels in blood at early time points are consistent with the increased hepatic clearance of rFVIIIFc in FVIII/VWF-DKO mice (Fig 2C and 2E) compared to FVIII-KO mice (Fig 2B and 2D). Interestingly, QWBA also reveals extensive radiolabel in the hepatic secretory pathway (bile, intestine) suggesting that ^125^I-rFVIIIFc is degraded in the liver of both FVIII-KO and FVIII/VWF-DKO mice (S1 Fig and S3–S6 Tables).

### Multiple cell types in liver express FcRn and various scavenger receptors

To determine the cells types in liver that may be capable of uptake and recycling of rFVIIIFc, we utilized qPCR to survey FcRn expression in freshly isolated liver cells (Table 1), since immunohistochemistry of endogenous FcRn in murine tissues is inconclusive [15,52]. We found that FcRn (*Fcgrt* mRNA) is expressed in major somatic cell types in the liver, such as hepatocytes and liver sinusoidal endothelial cells (LSEC), and in hematopoietic Kupffer cells. The purity of the isolated cells was confirmed by qPCR for specific markers including FVIII [17], stabilin-2 and Lyve-1 [53] for LSEC, CD68 and F4/80 (Erm-1) [54] in Kupffer cells, and albumin in hepatocytes. Similar analysis of reported scavenger receptors for VWF or FVIII [29–31,34–36] showed that many are expressed in multiple cell types in the liver (Table 1), suggesting redundancy in scavenger receptor mediated uptake.

**Table 1:** Expression of FcRn and candidate clearance receptors for FVIII and VWF in mouse liver.

Gene	Hepatocyte	LSEC	Kupffer Cell
**FcRn, VWF and endogenous FVIII**			
Fcgrt (FcRn)	21	100	26
F8 (FVIII)	0	100	3
VWF	0	100	4
**Clearance Receptors**			
LRP1	64	36	100
LDLR	100	22	26
Asgr1	100	1	3
Stabilin-2	0	100	3
Scara-5	100	3	11
Siglec-5	0	44	100
**Cell Type Markers**			
Albumin	100	2	3
Lyve-1	0	100	4
Emr-1 (F4/80)	0	34	100
CD68	3	25	100

Expression levels were determined by qPCR of mRNA in hepatocytes, LSEC and Kupffer cells purified from mouse livers (n = 2). For each gene, the highest ΔCt value is set at 100 and the ΔCt of that gene determined in the other two cell types are listed as a percentage of the highest ΔCt. The relative ΔCt values are derived from the mean of two replicates. The IgG recycling receptor FcRn (*Fcgrt*) is expressed in LSEC, hepatocytes, and Kupffer cells. Both FVIII and VWF mRNA is found to be enriched in the LSEC. Expression of potential scavenger receptors for VWF and FVIII in liver cells is variable and not always cell-specific. Cell-specific expression marker genes include albumin in hepatocytes, Lyve-1 in LSEC and CD68 and F4/80 (Emr-1) in Kupffer cells.

### FcRn in somatic cells not hematopoietic cells prolongs rFVIIIFc half-life

To determine the contribution of FcRn expressed in either hematopoietic (Kupffer cells and macrophages) or somatic (hepatocytes and LSEC) cells to the half-life prolongation of rFVIIIFc, we generated FcRn chimeric mice by bone marrow transplants using FcRn knock-out and isogenic wild-type mice. Flow cytometry of peripheral blood confirms >93 to 100% chimerism prior to the PK studies and immunohistochemical staining of liver cryosections for Kupffer cells (CD68). CD45.1 and CD45.2 isogenic markers confirm a high percent chimerism in liver Kupffer cells (S2 Fig).

**Fig 3:**
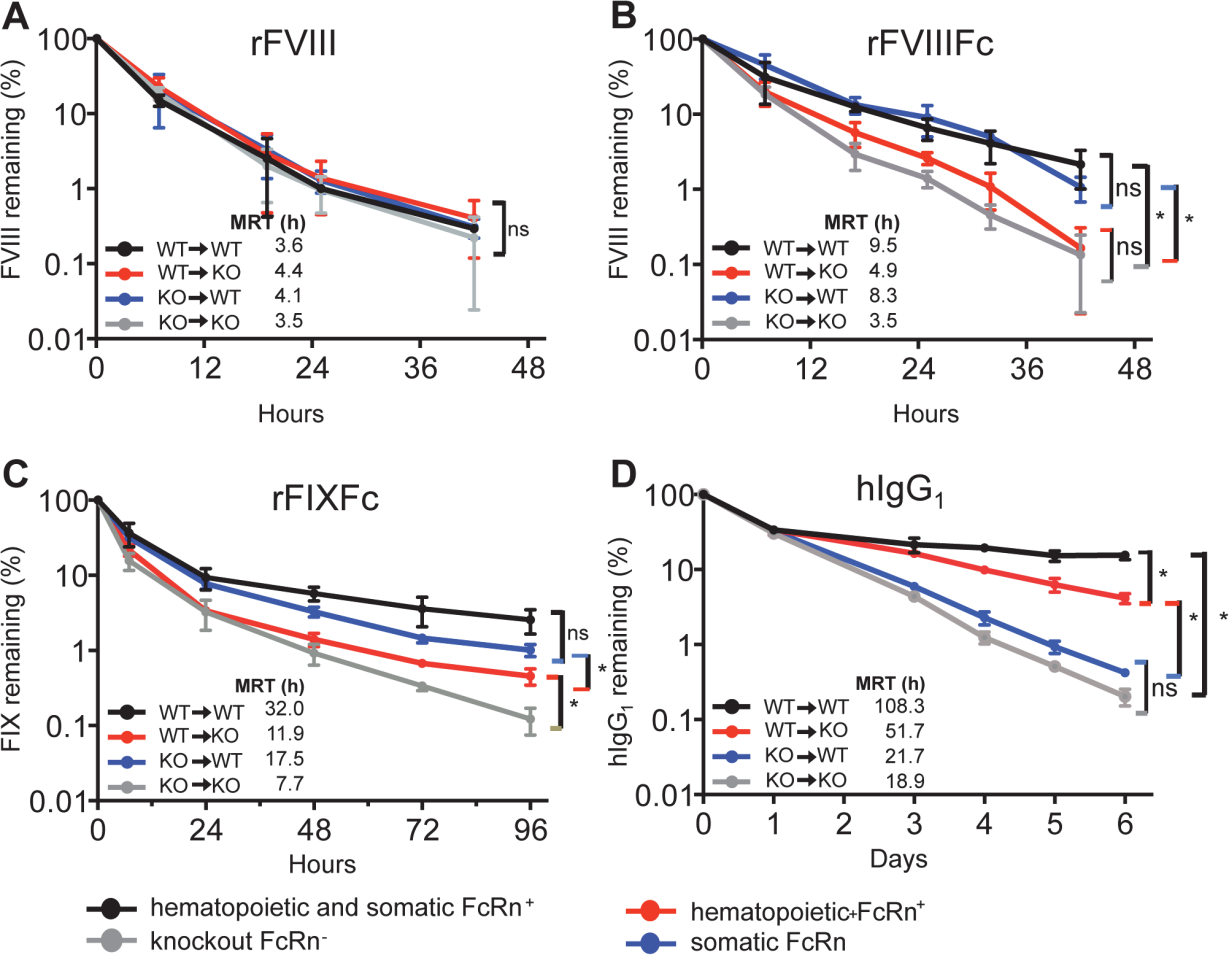
The half-life of rFVIIIFc is predominantly extended by FcRn expressed in somatic cells, but not in hematopoietic cells. The clearance of the following proteins is determined in bone marrow FcRn chimeric mice. (A) rFVIII (250 IU/kg), which clearance is not affected by FcRn (B) rFVIIIFc (250 IU/kg) which is predominantly protected from clearance by FcRn expressed in somatic cells (KO→WT). (C) rFIXFc (250 IU/kg) is mostly protected from clearance by FcRn expressed in somatic cells (KO→WT) (D) human IgG1 (5 mg/kg) is protected from clearance by FcRn expressed in hematopoietic cells (WT→KO). Plasma levels measured at 5 minute are set to 100% and the calculated mean residence times (MRT) are show in the graph insert, n = 4 for each data-point. Significance between plasma levels for individual time points on the PK curves is determined by an unpaired 2-tailed student t-test. A significant difference is indicated between PK curves with one or more significant time point differences (p<0.05).

We examined the pharmacokinetics of rFVIIIFc in these FcRn-chimeric mice using rFVIII as a control, as its half-life is unaffected by differences in FcRn expression. As expected, rFVIII shows similar clearance in all four chimeras (Fig 3A). In contrast, the half-life of rFVIIIFc is significantly extended 1.5-fold in both wild-type chimeric mice (WT→WT, FcRn expressed in all tissues) and in chimeric mice expressing FcRn only in somatic cells (KO→WT) (Fig 3B). rFVIIIFc is cleared rapidly in FcRn knock-out mice (KO→KO) similarly to rFVIII. FcRn expressed in hematopoietic cells (WT→KO) minimally prolongs the half-life of rFVIIIFc, excluding any significant contribution of Kupffer cells to the extended half-life of rFVIIIFc. Plasma levels of VWF are invariant among the chimeras, excluding VWF-mediated effects on the clearance of rFVIIIFc (S3 Fig).

The predominant contribution of somatic FcRn to the half-life extension is specific to rFVIIIFc as shown by control studies with rFIXFc and IgG1. Both somatic and hematopoietic FcRn contribute to the prolonged half-life of rFIXFc, although rFIXFc is protected to a greater extent by somatic rather than hematopoietic cell FcRn (Fig 3C). Human IgG1 has a longer half-life in FcRn wild-type mice than in FcRn knock-out mice [13,43] and hematopoietic FcRn makes a markedly greater contribution than somatic FcRn to the decreased clearance of IgG1 (Fig 3D). We further confirmed that wild-type mice have the highest endogenous levels of murine IgG1, while FcRn knock-out mice have the lowest, as previously reported [15]. Mice expressing either somatic or hematopoietic FcRn have intermediate endogenous IgG1 levels (S3 Fig).

### Immunohistochemistry of rFVIIIFc and rFVIII in livers of FVIII-KO and FVIII/VWF-DKO mice indicates both VWF-dependent and VWF-independent clearance pathways

To determine which cell types in liver are responsible for the clearance of rFVIIIFc and how cellular distribution is affected by VWF, we compared localization of rFVIIIFc and rFVIII in the presence or absence of VWF by immunohistochemistry. First, several fixation methods and dosing amounts were tested to optimize FVIII detection, using a panel of monoclonal antibodies specific to human FVIII and markers for LSEC (CD31), Kupffer cells (CD68) and VWF. To enhance detection of immunofluorescent signals, 4 monoclonal antibodies recognizing epitopes on different domains of FVIII were pooled [55]. In addition, mice received equimolar doses of 2.2 nmol/kg (~400 μg/kg) or 50- to 100-fold above the physiological plasma levels of FVIII resulting in between a 1:1 to 1:2 molar ratio of rFVIII or rFVIIIFc compared to endogenous plasma VWF monomer. Attempts to dose mice with 5-fold lower amounts of protein result in no detectable FVIII staining in livers (data not shown). Histology on organs harvested at time points later than 5 minutes post-dosing showed a rapid decrease in immunofluorescence signal at 30 minutes post-dosing in FVIII-KO and at 20 minute in FVIII/VWF-DKO mice (S4 Fig). This rapid loss of rFVIII or rFVIIIFc capable of binding antibodies that recognize intact protein suggests that much of the radioactivity beyond the 5 minute time point in Fig 2 is contributed by partially degraded protein in the liver. Staining controls, including control antibodies and stained sections of non-dosed animals, are included for each experiment as shown in S5–S7 Figs In studies with rFVIIIFc, results using FVIII antibodies were confirmed by staining with antibodies to the Fc domain of human IgG (S8 Fig).

**Fig 4:**
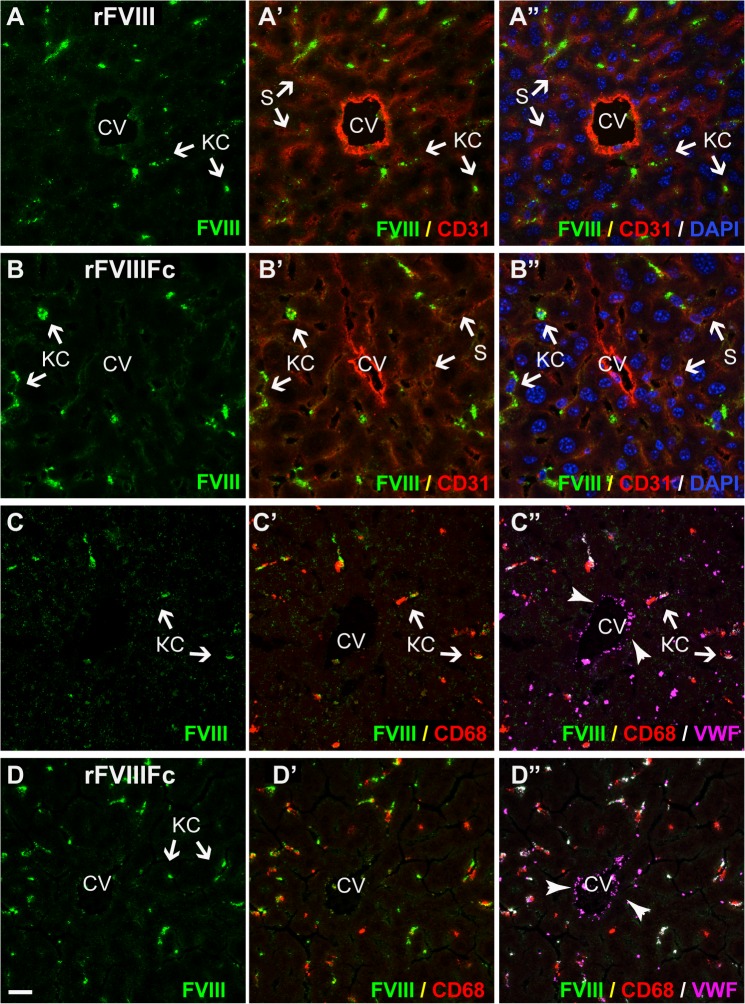
In FVIII-KO mice, both rFVIII and rFVIIIFc predominantly co-localize with VWF in liver Kupffer cells. In liver sections from FVIII-KO mice staining of both rFVIII (A and C, green) and rFVIIIFc (B and D, green) is intensely localized to Kupffer cells. In addition, rFVIII, but not rFVIIIFc, shows a distinct punctate staining associated with hepatocytes. Staining for LSEC and endothelium (A’ and B’, CD31, red) reveals the central vein (CV) and the diffuse sinusoidal network and confirms that the punctate staining of rFVIII (A’, green) is not associated with LSEC, while some rFVIIIFc staining (B’, green) localizes with the liver sinusoid. Nuclei are stained blue with DAPI (A’ and B”). Additional stainings confirm co-localization of rFVIII (C’) and rFVIIIFc (D’) in Kupffer cells (CD68, red). VWF (C” and D”, magenta) is also associated with FVIII staining in Kupffer cells (arrows), however VWF in Weibel-Palade bodies in the endothelial lining of the central vein is not associated with FVIII. For orientation in liver lobules: CV, central vein; S, sinusoid; KC, Kupffer cell and HC, plate of hepatocytes (scale bar, 20 μm).

**Fig 5:**
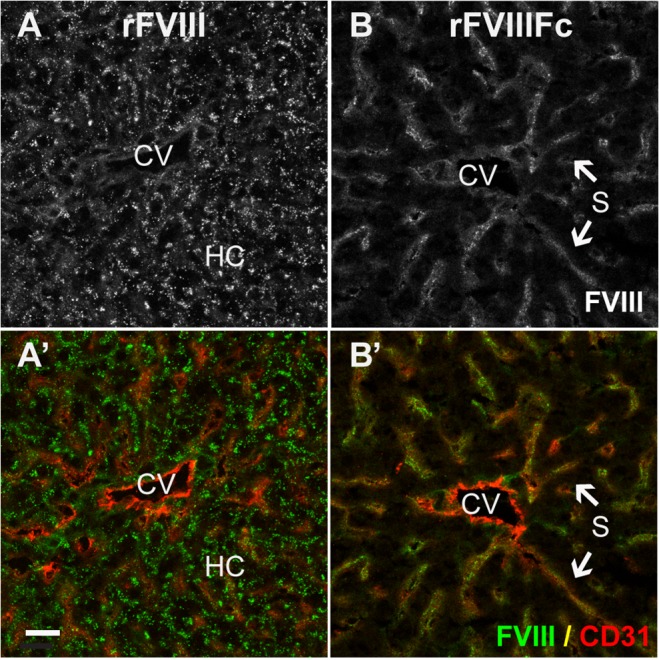
In the absence of VWF, rFVIII localizes to hepatocytes and rFVIIIFc is found in the liver sinusoid. Sections from FVIII/VWF-DKO mice lacking both VWF and FVIII, show strong punctate vesicular staining of rFVIII (A, A’, green) associated with hepatocytes, but not LSEC. In contrast, rFVIIIFc (B, B’, green) stains the liver sinusoid. Staining for LSEC and endothelium (A’, B’, CD31, red) confirms that the punctate staining of rFVIII is localized to the hepatocyte plate, while rFVIIIFc co-stains with the diffuse sinusoidal network of LSEC. (CV, central vein; S, sinusoid and HC, plate of hepatocytes; scale bar, 20 μm).

**Fig 6:**
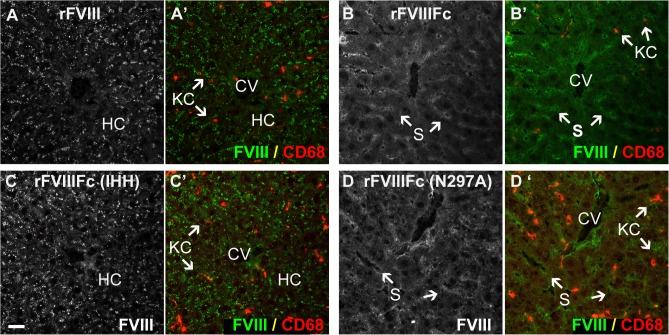
Distinct localization patterns for FcRn and FcRγ binding mutants of rFVIIIFc in the absence of VWF. Sections from FVIII/VWF-DKO mice lacking both VWF and FVIII, stained for Kupffer cells (A’, B’, C’, D’, CD68, red) show strong punctate vesicular staining of both rFVIII (A, A’, green) and the rFVIIIFc-IHH mutant (C, C’, green) that is incompetent to bind FcRn. This punctate staining is associated with hepatocytes and not LSEC. In contrast, both rFVIIIFc (B, B’, green) and the Fcγ-receptor binding mutant, rFVIIIFc-N297A (D, D’ green) localize to the liver sinusoid. In the absence of VWF, rFVIII, rFVIIIFc and the rFVIIIFc mutants are not associated with Kupffer cells (CD68, red). (CV, central vein; S, sinusoid; KC, Kupffer cell and HC, plate of hepatocytes; scale bar, 20 μm).

In FVIII-KO mice with endogenous VWF, both rFVIIIFc and rFVIII are found predominantly in Kupffer cells (Fig 4A–4D, and S4–S8 Figs), which also stain strongly for VWF (Fig 4C and 4D). In contrast, neither rFVIIIFc nor rFVIII is detected in large vessel endothelial cells that stain strongly for VWF present in Weibel-Palade bodies. Similarly, rFVIIIFc and rFVIII were detected in marginal zone macrophages in the spleen in the presence of endogenous VWF, but not in FVIII/VWF-DKO mice (S9 Fig). Close examination of liver sections reveals faint vesicular staining by rFVIII in hepatocytes (Fig 4C), in contrast to a diffuse staining of rFVIIIFc in the liver sinusoid (Fig 4D) in FVIII-KO mice. In FVIII/VWF-DKO mice lacking VWF (Figs 5 and 6A and 6B), neither rFVIII nor rFVIIIFc is detected in Kupffer cells, consistent with a VWF-dependent internalization of FVIII by these cells. In the absence of VWF, the majority of rFVIII is found in a vesicular staining pattern in hepatocytes. In contrast, rFVIIIFc again appears as a diffuse liver sinusoidal staining in the Space of Disse or in association with LSEC, in a staining pattern that is more prominent than in mice expressing VWF.

Since free rFVIII appears to be internalized by hepatocytes, we wondered whether free rFVIIIFc also enters hepatocytes before being cycled out of hepatocytes to localize in the liver sinusoid. We generated an Fc mutant of rFVIIIFc (rFVIIIFc-IHH) that is unable to bind FcRn. Similar to rFVIII, rFVIIIFc-IHH was detected only in hepatocytes in FVIII/VWF-DKO mice (Fig 6A and 6C), while in FVIII-KO mice expressing endogenous VWF, rFVIIIFc-IHH was found predominantly in Kupffer cells (data not shown), confirming the role of FcRn in rescue of VWF-free rFVIIIFc from hepatocytes. In contrast, rFVIIIFc-N297A, an rFVIIIFc mutant unable to bind FcγR, localized in the liver sinusoid similarly to rFVIIIFc in FVIII/VWF-DKO mice (Fig 6B and 6D), excluding a role for FcγR, which is expressed abundantly on LSEC, to target rFVIIIFc to the liver sinusoid.

## Discussion

Observations in patients with type 2N or type 3 von Willebrand disease, where binding of VWF to FVIII is impaired or VWF is absent, show that a loss of VWF binding results in decreased plasma levels of FVIII [56]. Similarly, the biodistribution studies reported here with radiolabeled rFVIIIFc in FVIII-KO and FVIII/VWF-DKO mice confirm that VWF stabilizes rFVIIIFc in circulation. Liver plays a major role in the clearance of clotting factors including FVIII and VWF [37,57], FVIIa [58] and FIX [59,60], in agreement with our observation that liver is the predominant clearance organ for rFVIIIFc. In the absence of VWF, 3-fold more ^125^I-rFVIIIFc was recovered in liver than when VWF is present.

The half-lives of both rFVIII and rFVIIIFc are greatly decreased in the absence of VWF, and in the absence of VWF the true efficiency of cycling by FcRn to protect rFVIIIFc from degradation is unmasked. Thus, we find a 5-fold difference in half-life between free rFVIIIFc and rFVIII in the absence of VWF in contrast to a 1.8-fold difference in the presence of VWF (Fig 1). These observations indicate that, although VWF is beneficial for the absolute maximal half-life obtained for rFVIIIFc [26], the clearance of the VWF-FVIII complex limits the maximum achievable half-life prolongation of for the FVIII/VWF complex, an observation also recently reported for glycopegylated FVIII [49].

Liver Kupffer cell uptake of the rFVIII-VWF complex has been reported previously [37,61]. We initially hypothesized that the clearance of rFVIIIFc was coupled to the clearance of VWF, and that rFVIIIFc may be rescued by FcRn in Kupffer cells and cycled back into circulation. To test this model, we depleted Kupffer cells and macrophages using clodrosomes (S1 Material and Methods). However, acute clodrosome treatment appears to affect liver function and LSEC activity, and the clearance of both rFVIIIFc and rFVIII is no longer coupled to VWF. Clodrosome treatment led to an increase in endogenous VWF, consistent with the decreased clearance of dosed murine plasma VWF in FVIII/VWF-DKO mice. In contrast, clodrosome treatment in both FVIII-KO and FVIII/VWF-KO resulted to an increased clearance of rFVIII and rFVIIFc (data not shown). Immediately following clodrosome treatment, the immunohistochemical staining of endogenous VWF shifted from the absent Kupffer cells and resulted in a strong sinusoidal staining (S10 Fig).

Rather than risk undefined effects due to an altered liver sinusoid resulting from the acute chemical depletion of macrophages, we turned to bone marrow transplant studies with FcRn knock-out mice to selectively remove FcRn from liver Kupffer cells and macrophages. Our chimeric FcRn mouse model shows that rFVIIIFc is protected predominantly by somatic cells expressing FcRn, indicating that the FcRn expressed in either hepatocytes or LSEC, but not in Kupffer cells, is responsible for cycling rFVIIIFc back into circulation following cellular uptake. These observations do not support our initial hypothesis that the uptake of rFVIIIFc and the VWF complex by liver Kupffer cells is followed by FcRn-mediated rescue of rFVIIIFc in Kupffer cells and excluded a major role of liver Kupffer cells or macrophages in the cycling of rFVIIIFc.

Our studies in FcRn chimeric mice also indicate that the cell type responsible for the cycling of Fc-fusion proteins is dictated by the protein, not the Fc domain. While rFVIIIFc is recycled predominantly by FcRn in somatic cells, we find a dominant role for FcRn in hematopoietic cells relative to somatic cells in the salvage of circulating IgG1, consistent with recent reports [9,13]. In addition, another coagulation factor Fc fusion protein, rFIXFc, is protected to a greater extent by somatic FcRn expressing cells than by hematopoietic cells. While FcRn is expressed in a variety of cells, it can only be available to rescue Fc fusion proteins that enter a specific cell type. These observations are consistent with studies in which IgG mutants with modified recognition to cellular epitopes but normal FcRn binding showed altered IgG clearance [7,62].

In order to visualize cells associated with rFVIII and rFVIIIFc by immunohistochemical staining it is necessary to dose both molecules at higher than the physiological level of FVIII. FVIII is a very potent cofactor in coagulation and circulates in blood at low molar levels of 0.5 to 1 nM. FVIII replacement therapies aim for similar low blood levels, which are very low compared to other therapeutic proteins, such as therapeutic antibodies where molar blood concentrations often are >1000-fold higher. The dose of rFVIII and rFVIIIFc used for immunohistochemical studies (5000 IU/kg) is 50- to 100-fold higher than that the dose needed to achieve normal FVIII levels (50 IU/kg). Published doses used for PK studies in mice (200–400 IU/kg) [47–49,63] range from 4- to 8-fold higher than the dose required to normalize plasma levels of FVIII activity. While the dose used for immunohistochemical studies is 12.5- to 25-fold higher than the doses used in published PK studies. The results of the immunohistochemical studies in this report are in good agreement with the biodistribution studies and PK studies in chimeric FcRn mice at lower doses of rFVIII and rFVIIIFc.

**Fig 7:**
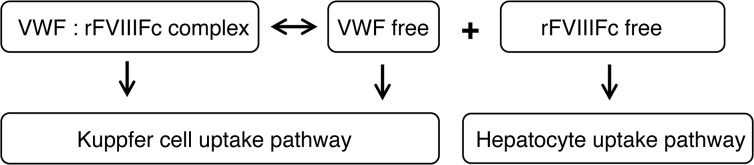
Scheme for two parallel uptake pathways in liver.

**Fig 8:**
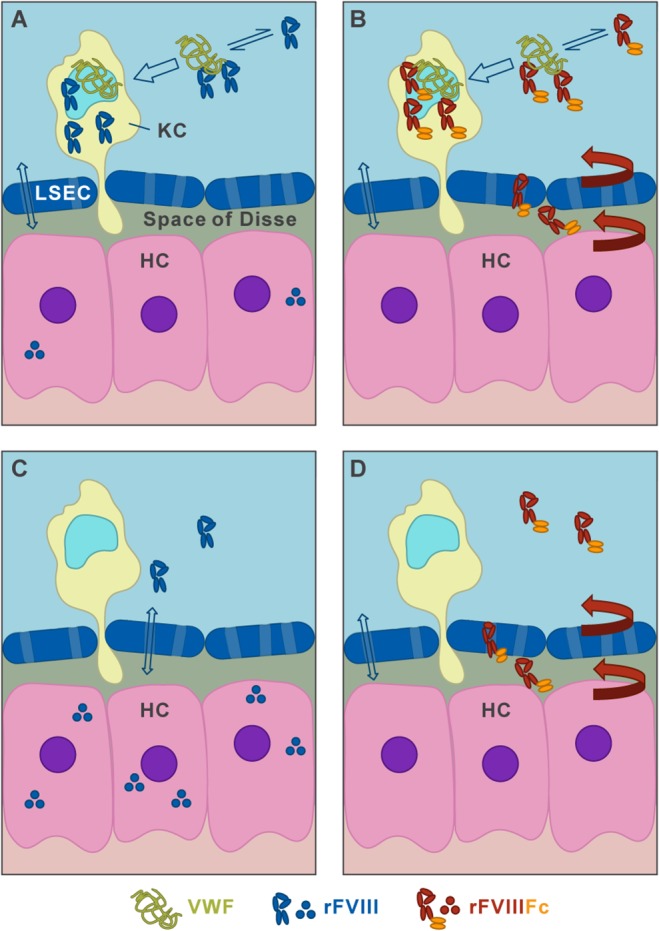
Model of two parallel liver clearance pathways for VWF-bound rFVIIIFc and free rFVIIIFc. In the presence of the dominant FVIII clearance determinant, VWF (A, B), the majority of either rFVIII (A) or rFVIIIFc (B) is in a dynamic complex with VWF (double black arrow). This FVIII/VWF complex is mainly cleared by hematopoietic derived Kupffer cells (KC, open blue arrow), where rFVIIIFc is not recycled by FcRn to prevent degradation. In contrast, VWF-free FVIII (C) or FVIIIFc (D), found either in the absence of VWF or transiently dissociated from the VWF complex, can enter hepatocytes (HC) after diffusion through fenestrae (light blue with open double arrow). While rFVIII is degraded in hepatocytes, free rFVIIIFc entering hepatocytes is cycled by FcRn (red arrows) into the Space of Disse lined by LSEC. This leads to the liver sinusoidal staining pattern observed for rFVIIIFc in FVIII/VWF-DKO mice and allows it to reenter circulation, thereby improving the half-life of FVIIIFc.

Based on our findings in this report, we propose a model with two parallel pathways for the clearance and cycling of rFVIIIFc shown schematically in Fig 7 and illustrated Fig 8. As shown in Fig 7, a dynamic binding equilibrium between FVIII and VWF always results in both FVIII in complex with VWF and a small fraction of FVIII that is free of VWF. The major determinant of FVIII half-life is a pathway in which molecules of rFVIII or rFVIIIFc complexed with VWF are stabilized and protected from rapid clearance. Ultimately, this FVIII complexed by VWF is internalized and degraded by liver Kupffer cells and macrophages. This pathway is in agreement with published studies in which rFVIII in complex with VWF is shown to be internalized by liver Kupffer cells [37]. However, our studies in FcRn-chimeric mice exclude a role for hematopoietic-derived cells in the FcRn-mediated salvage of rFVIIIFc, indicating that rFVIIIFc in complex with VWF cannot be recycled by FcRn in Kupffer cells, which is consistent with the observation that the overall half-life extension of rFVIIIFc is unable to exceed that of VWF [3,4].

Our finding of a second clearance pathway in which both free rFVIII and free rFVIIIFc can be internalized by liver hepatocytes is novel. Free rFVIII is retained in hepatocytes, while free rFVIIIFc that enters hepatocytes is cycled by FcRn back to the liver sinusoid and into circulation. Our immunohistochemical data shows that free rFVIII is retained in hepatocytes, while free rFVIIIFc is predominantly associated with liver sinusoids. In circulation, the majority of rFVIII and rFVIIIFc binds the large multimeric VWF (500–20,000 kD) [64]. The VWF multimer may extend up to 100 μm in length [65,66]. We speculate that the size of VWF/rFVIII (or rFVIIIFc) complex limits its entry into the Space of Disse through the sinusoidal fenestrae (150 nm), and subsequently, the access to hepatocytes [67–69]. However, because complex formation between VWF and rFVIIIFc or rFVIIIFc is in dynamic equilibrium [31,70,71], a fraction of rFVIII or rFVIIIFc will always be free and available for uptake and clearance by hepatocytes. Indeed, based on qPCR analysis we found that hepatocytes express several scavenger receptors that have been implicated in the clearance of FVIII, includingLRP1, LDLR, Asgr1 and Scara-5. Several of these and additional receptors are also expressed on LSEC and Kuppfer cells (Table 1). Free rFVIIIFc entering hepatocytes is cycled by FcRn back to the liver sinusoid and circulation, while free rFVIII or the rFVIIIFc-IHH mutant entering hepatocytes cannot be rescued by FcRn.

Our findings and our model raise several important questions. Immunohistochemical staining of rFVIIIFc in the liver sinusoid does not unequivocally discriminate between rFVIIIFc in the Space of Disse and rFVIIIFc associated with LSEC in this study. Therefore, we cannot exclude the possibility of additional cycling by FcRn expressed in somatic cell derived LSEC. However, since rFVIIIFc-IHH mutant that cannot be recycled by FcRn was found to accumulate in only hepatocytes not LSEC, the contribution of LSEC FcRn mediated recycling will be minor. In addition, it is unclear how low shear flow in the liver sinusoid may affect the ratio of free rFVIIIFc to rFVIIIFc in complex with VWF. Factors that increase the amount of free rFVIIIFc in the liver relative to that in complex with VWF in circulation may also impact the uptake and cycling of rFVIIIFc by hepatocytes.

In conclusion, our studies showed that VWF contributes to the reduced clearance by the liver of both rFVIII and rFVIIIFc, however binding to VWF is ultimately the major contributor to FVIII and FVIIIFc uptake in Kupffer cells and macrophages. In addition, we found that the VWF-free fraction of FVIII is degraded by hepatocytes, while free-rFVIIIFc is rescued from these somatic cells and recycled by FcRn, thereby prolonging the circulating plasma levels of rFVIIIFc.

## Supporting Information

S1 Fig QWBA images of rFVIIIFc in FVIII-KO and FVIII/VWF-DKO mice. Pseudo-color coded autoradiographs of rFVIIIFc in FVIII-KO mice deficient for factor VIII at (A-C) 5 and 30 minutes and 16 hours (~one half-life time) and FVIII/VWF-DKO mice deficient for both factor VIII and VWF at (D-F) 5 and 15 minutes and 1 hour (~one half-life time) post-dose. Pseudo-colored internal standards represents 3076, 1502, 368, 88.9, 21.8, 5.53, 1.36 and 0.338 ng ^125^I-rFVIIIFc/g tissue (top to bottom). The rapid accumulation of label in urine indicates glomerular clearance of ^125^I-rFVIIIFc degradation products (<3 to 8% of input dose at early time points, S3, S5 Tables). At later time points, the radiolabel in the bile and intestine (hepatic secretory pathway), indicating degradation of ^125^I-rFVIII/rFVIIIFc in the liver (S4, S6 Tables).
(TIF)

S2 Fig Assessment of chimerism in bone marrow transplant mice. (A) Cohorts of FcRn-chimeric mice used in studies (BMT 3–6) show 93% to 99.6% chimerism as determined by flow cytometry analysis of blood cells, using matching isogenic markers CD90.1 (WT) and Cd90.2 (KO) or Cd45.1 (WT) and CD45.2 (KO). The % chimerism ± SD (n = 10) was determined for each cohort. BMT5 mice did not receive an intermediate treatment with clodrosomes to remove radiation resistant Kupffer cells[46] and this did not appear to affect the chimerism of blood or liver cells. (B) Liver cell chimerism assessed by immunohistochemical co-staining with F4/80 Kupffer cell marker and isotype markers. (C) Quantitation of liver cell chimerism by immunohistochemical comparison of co-staining marker surface area in whole sections stained for specific Kupffer cell and isotype markers shows 60–90% chimerism in the low percentage (~3%) of Kupffer cell staining area (pseudocolors for cellular markers in B were assigned using Volocity imaging software to accommodate visual evaluation of triple co-staining signal).
(TIF)

S3 Fig Relative levels of endogenous VWF and IgG1 in FcRn chimeric mice. (A) Endogenous VWF plasma levels determined by ELISA do not differ between the chimeric groups (n = 5), excluding differences in VWF levels as a factor affecting the clearance of rFVIIIFc. **(**B) Relative serum levels of endogenous IgG_1_ in FcRn-chimeric mice. The highest levels of endogenous IgG1 are observed in wild-type mice and the lowest levels in FcRn-KO mice as reported previously[15]. Interestingly, both hematopoietic and somatic FcRn expressing cells contribute equally to reduced endogenous IgG1 levels.
(TIF)

S4 Fig rFIII and rFVIIIFc staining signal decreases with increasing post-dosing times in FVIII-KO and FVIII/VWF-DKO mice. FVIII-KO (A-F) or FVIII/VWF-DKO mice (G-J) were dosed with equimolar amounts of rFVIII (296 μg/kg) (A-C, G-H) or rFVIIIFc (484 μg/kg) (D-F, I-J). At different times post dosing, mice were sacrificed and cryosections prepared. (A, D, G, I) 5 minutes; (H, J), 20 minutes; (B,E), 30 minutes; (C), 4 hours and (D), 5 hours. Sections were stained using a primary antibody mixture against FVIII and CD68 (A-F) or FVIII and CD31 (G-J). In FVIII-KO mice (A-F), signal for both rFVIII and rFVIIIFc is detected in most Kupffer cells at 5 minutes and signal decreases over time to background 4–5 hours. In FVIII/VWF-DKO mice specific staining signal in hepatocytic vesicles (G) for rFVIII and sinusoids (I) of rFVIIIFc decreases to background levels within 20 minutes (H and J)/ Merges images for endothelial staining (CD31) G’-J’). For orientation: CV, central vein, scale bars, 20 μm.
(TIF)

S5 Fig Immunohistology of controls and rFVIII and rFVIIIFc costained for Kupffer cells and VWF in FVIII-KO mice. Mice were dosed with equimolar amounts of rFVIII (296 μg/kg) (A, D) or rFVIIIFc (484 μg/kg) (B) or nothing (naïve) (C). Five minutes post dosing mice were sacrificed and cryosections prepared, see material and methods. Sections were stained using identical staining conditions, primary antibody mixture against FVIII, CD68 and VWF (A-C) or CD68 and VWF (D), signal was detected using the identical secondary antibody mixture (anti-mouse-IgG2a-Alexa594, anti-rat-Alexa488 and anti-rabbit-Alexa647. All imaging capture and processing settings are identical. Panels A and B show FVIII signal mostly in Kupffer cells, while negative controls C and D lack staining signal. Merged images for Kupfer cells (CD68, A’-D’) and VWF (A” –D”) show VWF localized in Kupffer cells and endothelial cells aligning large blood vessels. FVIII signal colocalizes with CD68 and VWF in Kupffer cells. For orientation: CV, central vein; KC, Kupffer cell, scale bar, 20 μm.
(TIF)

S6 Fig Immunohistology of controls and rFVIII and rFVIIIFc costained for Kupffer cells and VWF in FVIII/VWF-DKO mice. Mice were dosed with equimolar amounts of rFVIII (296 μg/kg) (A, D) or rFVIIIFc (484 μg/kg) (B) or nothing (naïve) (C). Five minutes post dosing mice were sacrificed and cryosections prepared, see material and methods. Sections were stained using identical staining conditions, primary antibody mixture against FVIII, CD68 and VWF (A-C) or CD68 and VWF (D), signal was detected using the identical secondary antibody mixture (anti-mouse-IgG2a Alexa594, anti-rat-Alexa488 and anti-rabbit-Alexa647. All imaging capture and processing settings are identical. Panels A,A’ (rFVIII) and B,B’ (rFVIIIFc) show lack of FVIII signal from Kupffer cells (CD68, green), while negative controls in naïve mice C or dosed mice stained lacking primary anti-FVIII antibody (D) lack FVIII staining signal completely. rFVIII shows a vesicular staining in hepatocytes (A, A’), while rFVIIIFc shows a (patchy) sinusoidal staining pattern (B,B’). Merged images co-stained for Kuppfer cells (CD68) and VWF, show the complete lack of VWF signal, as expected in FVIII/VWF-DKO mice. For orientation: CV, central vein; HC, hepatocyte; KC, Kupffer cell; S, sinusoid. Scale bar, 20 μm.
(TIF)

S7 Fig Immunohistology of controls and rFVIII and rFVIIIFc costained for endothelial cells in FVIII-KO and FVIII/VWF-DKO mice. FVIII-KO (A-C) or FVIII/VWF-DKO mice (D-F) were dosed with equimolar amounts of rFVIII (296 μg/kg) (A, D) or rFVIIIFc (484 μg/kg) (B, E) or nothing (naïve, C, F). Five minutes post dosing mice were sacrificed and cryosections prepared, see material and methods. Sections were stained using identical staining conditions, primary antibody mixture against FVIII and CD31 signal was detected using the identical secondary antibody mixture (anti-mouse-IgG2a-Alexa488 and anti-rat-Alexa594. All imaging capture and processing settings are identical. In FVIII-KO mice (A-C), panels A and B show FVIII signal mostly in Kupffer cells, while no FVIII signal is detected in naïve mice C. A’-C’ are merged images for endothelial cell costaining (CD31). In FVIII/VWF-DKO mice (D-F) rFVIII signal is detected in hepatocytes (D, D’) and a fainter signal for rFVIIIFc is detected in sinusoids (E), costaining with endothelial cells (CD31, E’), no FVIII signal is detected in naïve sections of DKO mice (F, F’) For orientation: CV, central vein, scale bars, 20 μm.
(TIF)

S8 Fig Immunohistological detection using anti-Fc for rFVIIIFc and controls in FVIII-KO and FVIII/VWF-DKO mice. FVIII-KO (A-B, E-F) or FVIII/VWF-DKO mice (C-D, G-H) were dosed with equimolar amounts of rFVIII (296 μg/kg) (A-D) or rFVIIIFc (484 μg/kg) (E-H). Matched sections were stained using primary antibody mixture against FVIII and CD31 (A, C, E, G) or CD31 and human-IgG1 (B,D,F,H). In FVIII-KO mice gives anti-human Fc a Kupffer cell staining pattern for rFVIIIFc (F,F’) similar to that obtained using anti-FVIII (E). Negative control, is the lack of Kupffer cell signal in the anti-Fc staining (B) for rFVIII in as observed using anti-FVIII (A,A’). In FVIII/VWF-KO mice, sinusoidal staining of rFVIIIFc is detected by both anti-FVIII (G,G”) and Fc (H, H’). As a control, anti-Fc does not stain the strong hepatocyte vesicular pattern (D,D’) as observed for rFVIII in DKO mice (C,C’). Scale bars, 20 μm.
(TIF)

S9 Fig Internalization of rFVIII and rFVIIIFc by splenic macrophages depends on VWF. Both rFVIII (A-C) and rFVIIIFc (D-F) are predominantly internalized in MARCO+ (red) marginal zone (MZ) macrophages of the spleen (B, E) in FVIII-KO mice. VWF staining is absent in the metallophilic marginal zone macrophages stained with MOMA-1/CD169 (red) adjacent to the white pulp (WP) but instead extends from the FVIII and MARCO+ positive macrophages in the red pulp (RP) (A’,D’). rFVIII (C) and rFVIIIFc (F) staining (green) is still detected in a few marginal zone cells 4 and 5 hours after dosing. In FVIII/VWF-DKO mice neither rFVIII nor rFVIIIFc signal is detected in splenic macrophages at 5 minutes (not shown) similar to the VWF dependence of uptake by liver Kupffer cells. Original magnification is 200x.
(TIF)

S10 Fig Acute clodrosome treatment increases the sinusoidal localization of endogenous VWF in FVIII-KO mice, but decreases rFVIIIFc staining in Kupffer cells and liver sinusoid. One day after clodrosome treatment, rFVIIIFc, endogenous VWF and Kupffer cells were stained in liver sections of FVIII-KO mice prepared 5 minutes after dosing rFVIIIFc. Control livers (A-A”) were compared to Kupffer cell depleted livers (B-B”). In control liver, rFVIIIFc is detected predominantly in Kupffer cells (A), along with endogenous VWF, which is also detected in the Weibel-Palade bodies of the large blood vessels (A’). An overlay of staining for rFVIIIFc, VWF and CD68, a Kuppfer cell marker, is shown (A”). In clodrosome treated FVIII-KO mice the rFVIIIFc signal is almost undetectable in liver (B) and endogenous VWF is no longer concentrated in Kupffer cells, as expected, but VWF staining remains in the endothelial cells of the larger vessels (B’). In addition, a relatively large increase in VWF staining is observed in liver sinusoid following clodrosome treatment. Decreased and diffuse staining of the Kupffer cell marker CD68 only on cell fragments confirms that clodrosome treatment depletes liver Kupffer cells (B”). For orientation in liver lobules: CV, central vein; S, sinusoid; KC and Kupffer cell (scale bar, 20 μm).
(TIF)

S1 Material and Methods Clodrosome mediated Kupffer cell depletion (PDF)

S1 Table Antibodies used for immunohistochemistry and FACS. (PDF)

S2 Table LifeTechnologies qPCR primers for liver cell analysis. (PDF)

S3 Table Biodistribution of rFVIIIFc or rFVIII calculated as %ID/organ by QWBA or scintillation counting in FVIII-KO mice. (PDF)

S4 Table Percentage of injected dose per gram of tissue (%ID/g) for rFVIIIFc or rFVIII as determined by QWBA or scintillation counting FVIII-KO mice. (PDF)

S5 Table Biodistribution of rFVIIIFc as calculated %ID/organ as determined by QWBA or scintillation counting in FVIII/VWF DKO Mice. (PDF)

S6 Table Percentage of injected dose per gram of tissue (%ID/g) of rFVIIIFc as determined by QWBA or scintillation counting in FVIII/VWF DKO Mice (PDF)
