# Supplementary figures and images for: FcRn Rescues Recombinant Factor VIII Fc Fusion Protein from a VWF Independent FVIII Clearance Pathway in Mouse Hepatocytes
Source: PLoS One. 2015 Apr 23;10(4):e0124930. doi: 10.1371/journal.pone.0124930 (PMC4408089; doi:10.1371/journal.pone.0124930)

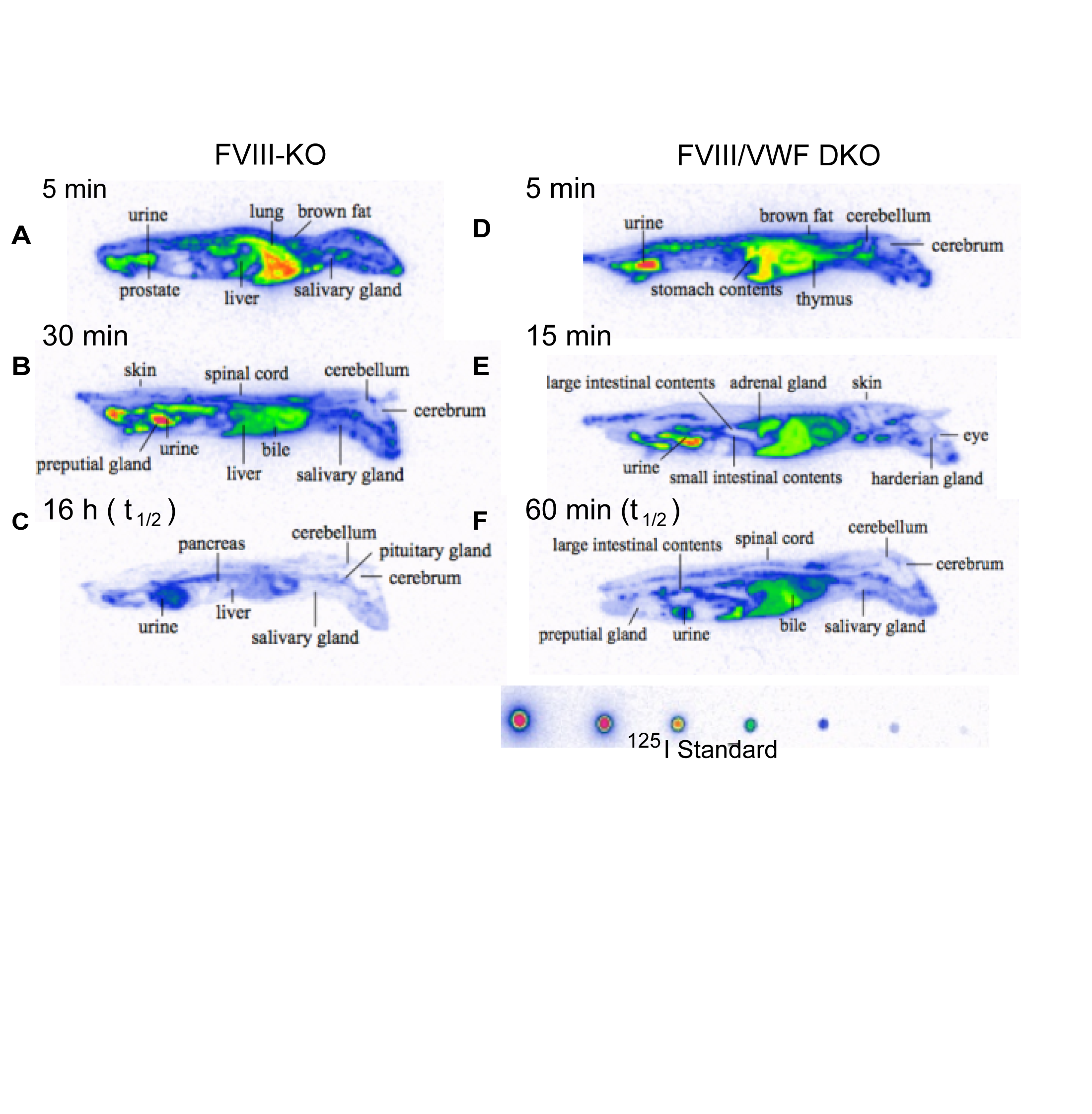

Supplement: S1 Fig — Pseudo-color coded autoradiographs of rFVIIIFc in FVIII-KO mice deficient for factor VIII at (A-C) 5 and 30 minutes and 16 hours (~one half-life time) and FVIII/VWF-DKO mice deficient for both factor VIII and VWF at (D-F) 5 and 15 minutes and 1 hour (~one half-life time) post-dose. Pseudo-colored internal standards represents 3076, 1502, 368, 88.9, 21.8, 5.53, 1.36 and 0.338 ng 125I-rFVIIIFc/g tissue (top to bottom). The rapid accumulation of label in urine indicates glomerular clearance of 125I-rFVIIIFc degradation products (<3 to 8% of input dose at early time points, S3, S5 Tables). At later time points, the radiolabel in the bile and intestine (hepatic secretory pathway), indicating degradation of 125I-rFVIII/rFVIIIFc in the liver (S4, S6 Tables). (TIF) [file pone.0124930.s001.tif]

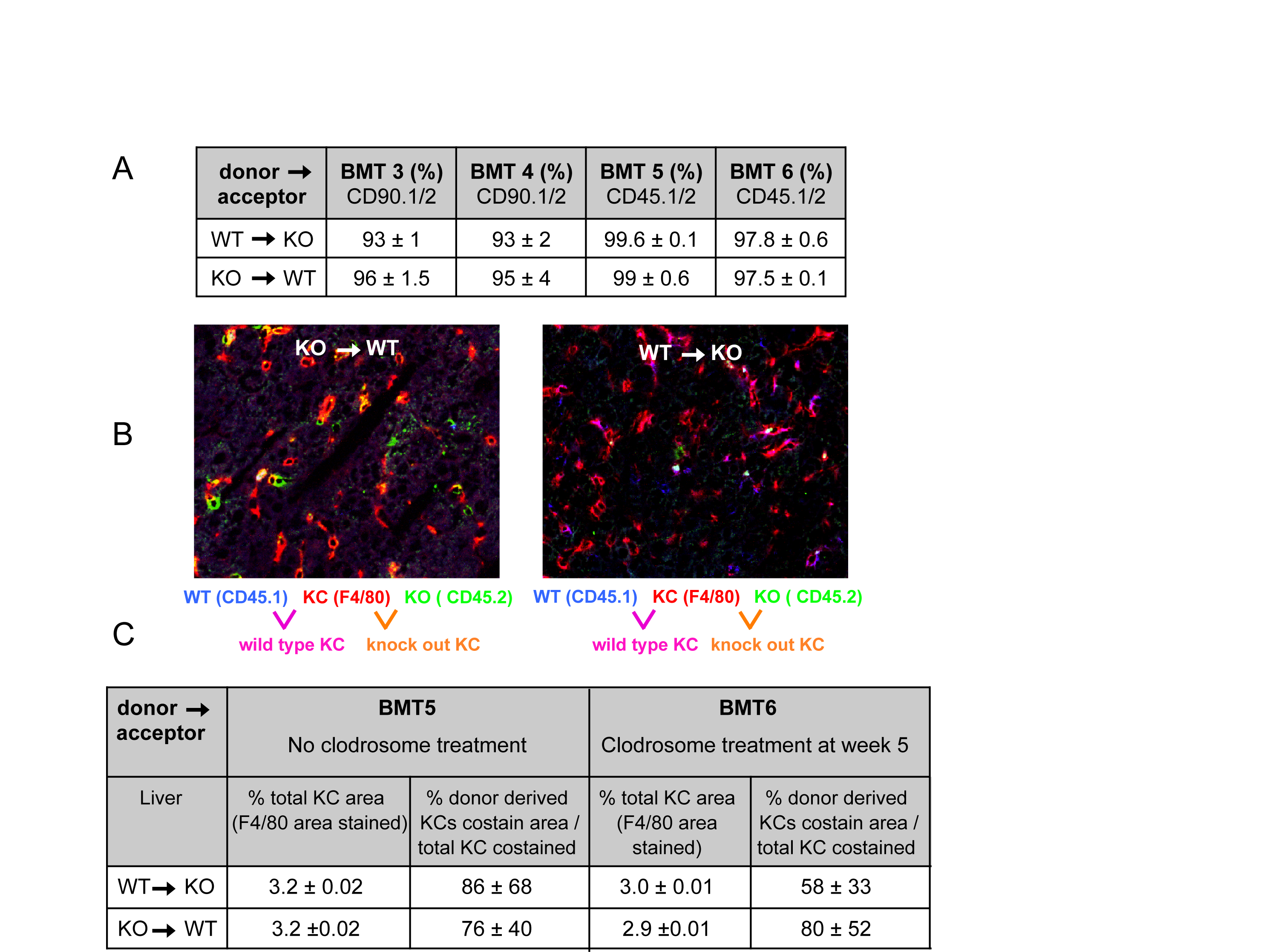

Supplement: S2 Fig — (A) Cohorts of FcRn-chimeric mice used in studies (BMT 3–6) show 93% to 99.6% chimerism as determined by flow cytometry analysis of blood cells, using matching isogenic markers CD90.1 (WT) and Cd90.2 (KO) or Cd45.1 (WT) and CD45.2 (KO). The % chimerism ± SD (n = 10) was determined for each cohort. BMT5 mice did not receive an intermediate treatment with clodrosomes to remove radiation resistant Kupffer cells[46] and this did not appear to affect the chimerism of blood or liver cells. (B) Liver cell chimerism assessed by immunohistochemical co-staining with F4/80 Kupffer cell marker and isotype markers. (C) Quantitation of liver cell chimerism by immunohistochemical comparison of co-staining marker surface area in whole sections stained for specific Kupffer cell and isotype markers shows 60–90% chimerism in the low percentage (~3%) of Kupffer cell staining area (pseudocolors for cellular markers in B were assigned using Volocity imaging software to accommodate visual evaluation of triple co-staining signal). (TIF) [file pone.0124930.s002.tif]

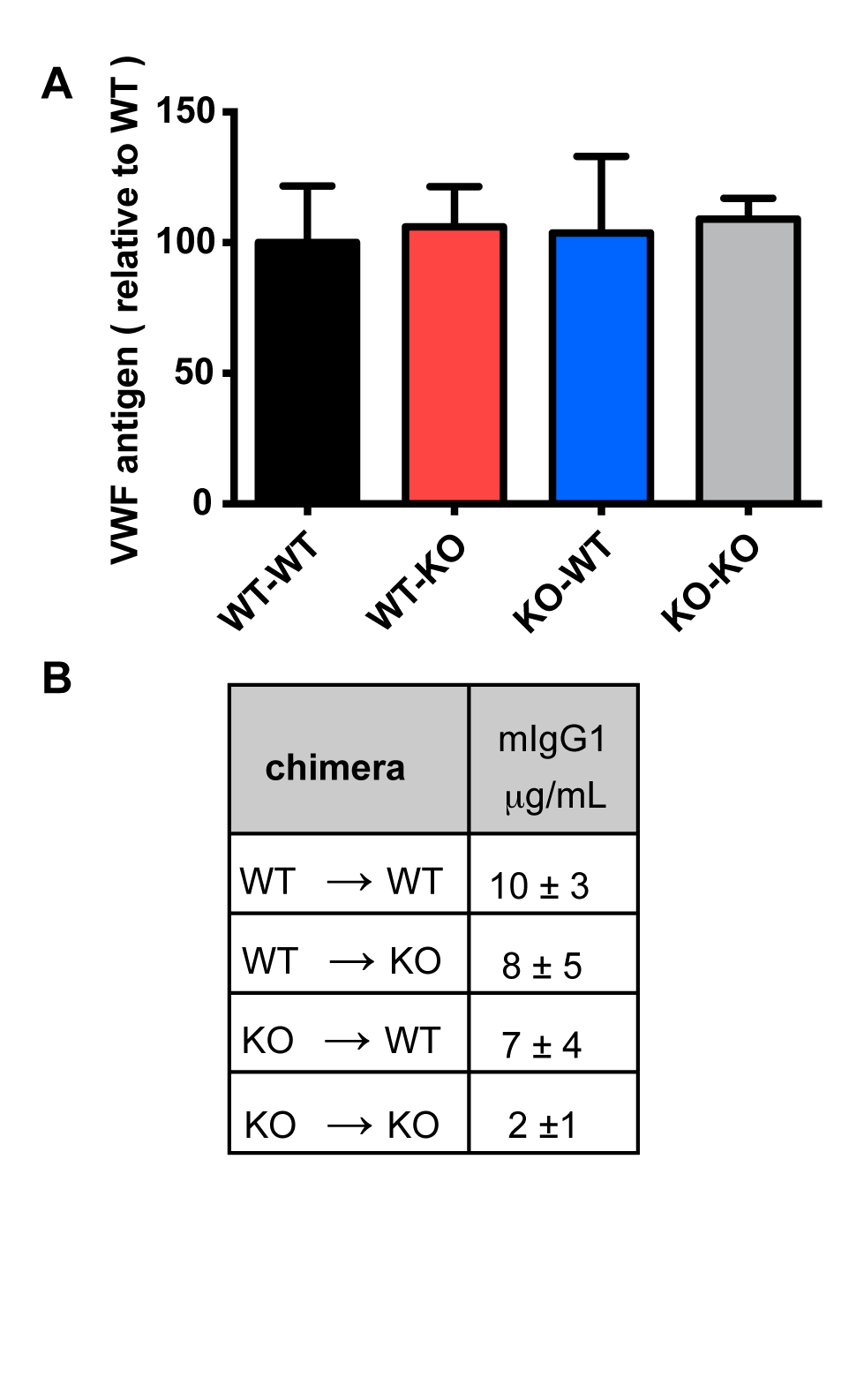

Supplement: S3 Fig — (A) Endogenous VWF plasma levels determined by ELISA do not differ between the chimeric groups (n = 5), excluding differences in VWF levels as a factor affecting the clearance of rFVIIIFc. (B) Relative serum levels of endogenous IgG1 in FcRn-chimeric mice. The highest levels of endogenous IgG1 are observed in wild-type mice and the lowest levels in FcRn-KO mice as reported previously[15]. Interestingly, both hematopoietic and somatic FcRn expressing cells contribute equally to reduced endogenous IgG1 levels. (TIF) [file pone.0124930.s003.tif]

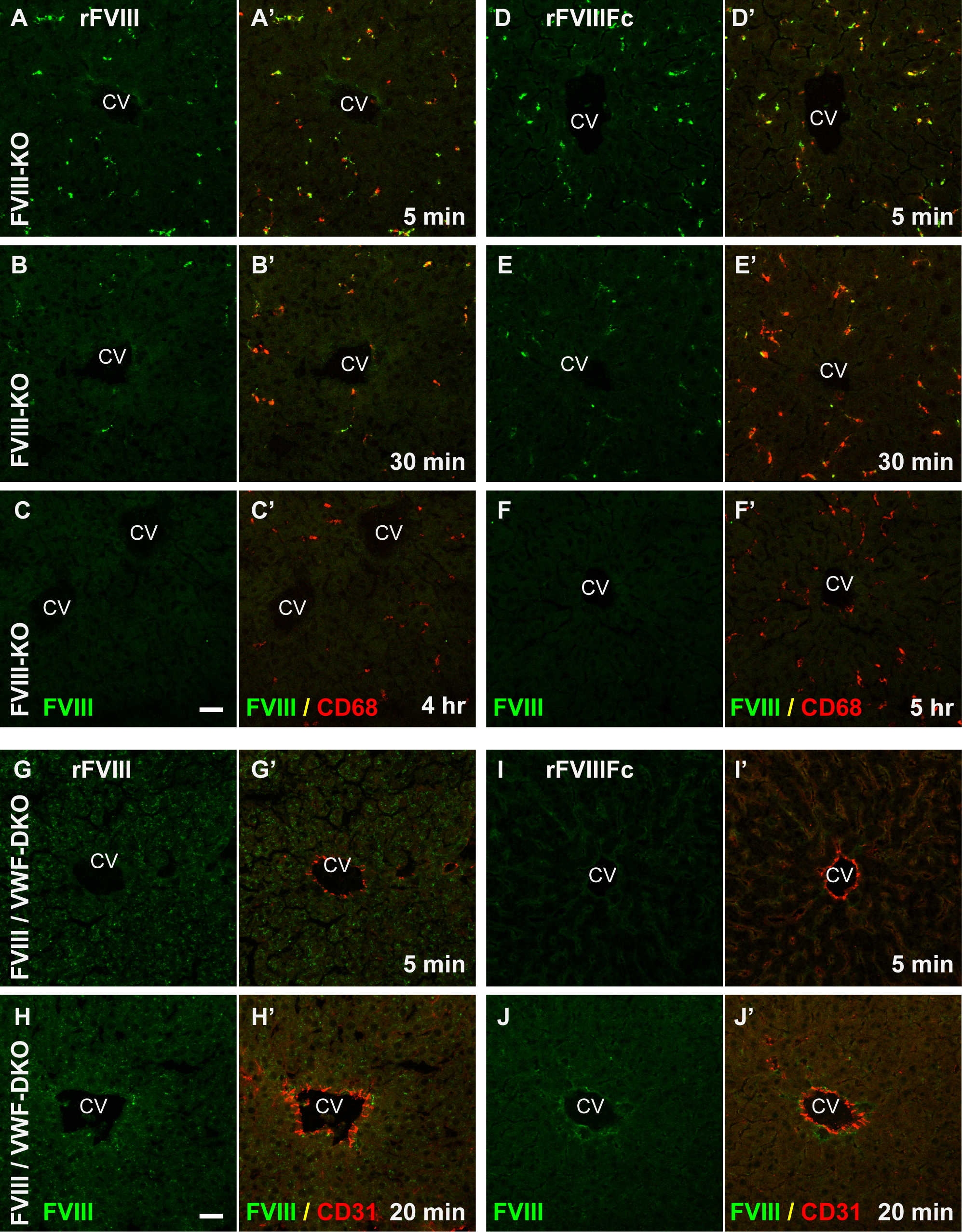

Supplement: S4 Fig — FVIII-KO (A-F) or FVIII/VWF-DKO mice (G-J) were dosed with equimolar amounts of rFVIII (296 μg/kg) (A-C, G-H) or rFVIIIFc (484 μg/kg) (D-F, I-J). At different times post dosing, mice were sacrificed and cryosections prepared. (A, D, G, I) 5 minutes; (H, J), 20 minutes; (B,E), 30 minutes; (C), 4 hours and (D), 5 hours. Sections were stained using a primary antibody mixture against FVIII and CD68 (A-F) or FVIII and CD31 (G-J). In FVIII-KO mice (A-F), signal for both rFVIII and rFVIIIFc is detected in most Kupffer cells at 5 minutes and signal decreases over time to background 4–5 hours. In FVIII/VWF-DKO mice specific staining signal in hepatocytic vesicles (G) for rFVIII and sinusoids (I) of rFVIIIFc decreases to background levels within 20 minutes (H and J)/ Merges images for endothelial staining (CD31) G’-J’). For orientation: CV, central vein, scale bars, 20 μm. (TIF) [file pone.0124930.s004.tif]

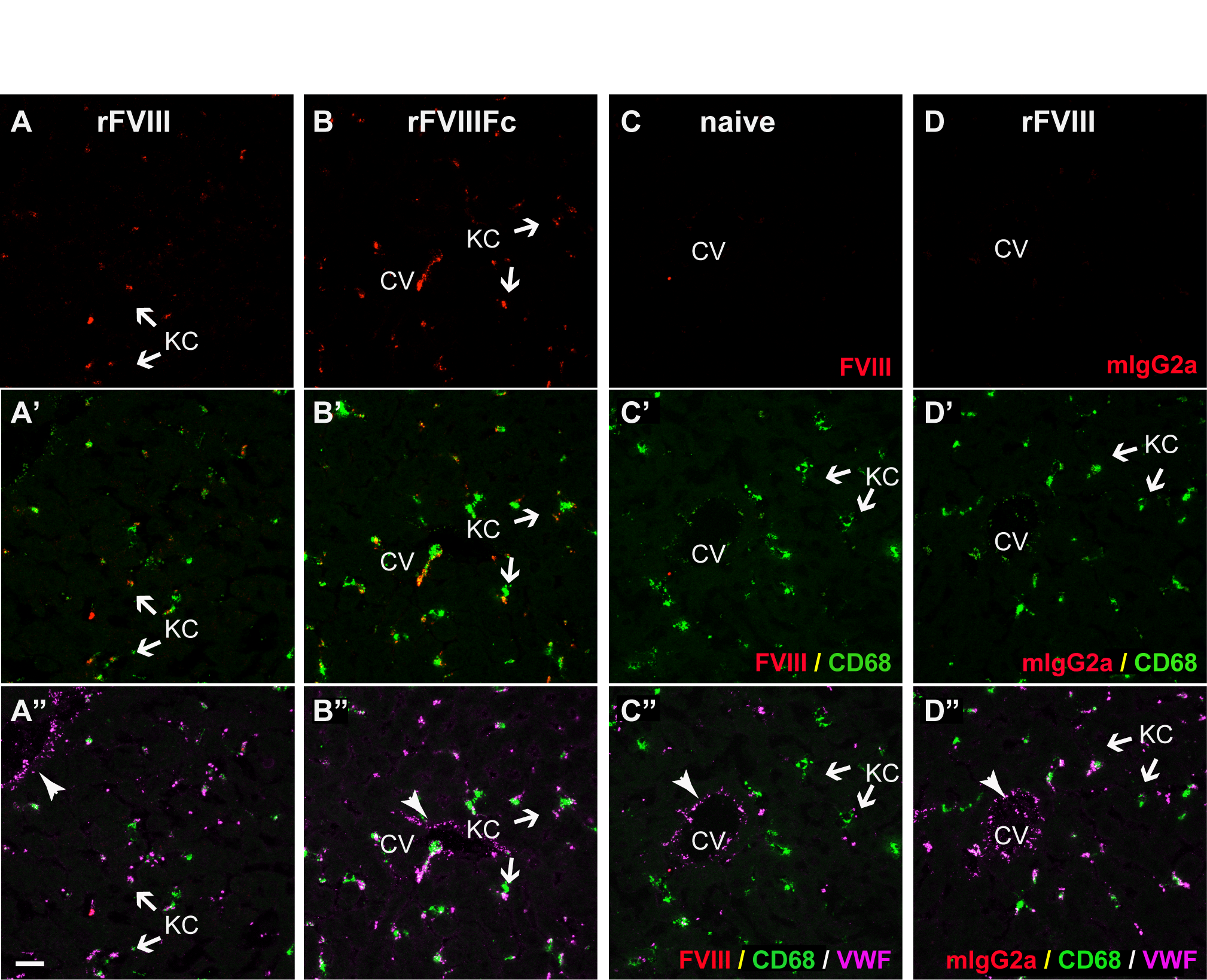

Supplement: S5 Fig — Mice were dosed with equimolar amounts of rFVIII (296 μg/kg) (A, D) or rFVIIIFc (484 μg/kg) (B) or nothing (naïve) (C). Five minutes post dosing mice were sacrificed and cryosections prepared, see material and methods. Sections were stained using identical staining conditions, primary antibody mixture against FVIII, CD68 and VWF (A-C) or CD68 and VWF (D), signal was detected using the identical secondary antibody mixture (anti-mouse-IgG2a-Alexa594, anti-rat-Alexa488 and anti-rabbit-Alexa647. All imaging capture and processing settings are identical. Panels A and B show FVIII signal mostly in Kupffer cells, while negative controls C and D lack staining signal. Merged images for Kupfer cells (CD68, A’-D’) and VWF (A” –D”) show VWF localized in Kupffer cells and endothelial cells aligning large blood vessels. FVIII signal colocalizes with CD68 and VWF in Kupffer cells. For orientation: CV, central vein; KC, Kupffer cell, scale bar, 20 μm. (TIF) [file pone.0124930.s005.tif]

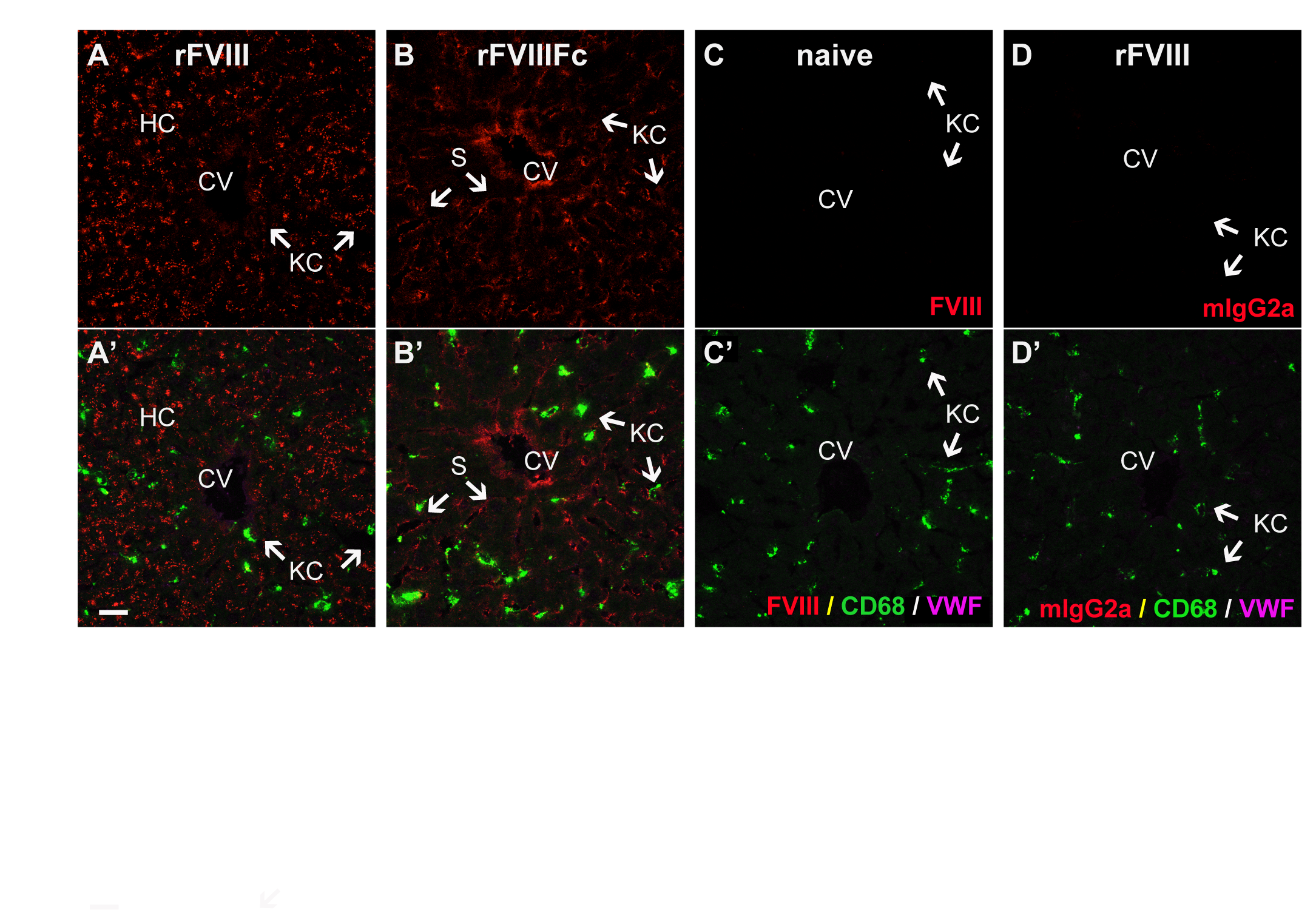

Supplement: S6 Fig — Mice were dosed with equimolar amounts of rFVIII (296 μg/kg) (A, D) or rFVIIIFc (484 μg/kg) (B) or nothing (naïve) (C). Five minutes post dosing mice were sacrificed and cryosections prepared, see material and methods. Sections were stained using identical staining conditions, primary antibody mixture against FVIII, CD68 and VWF (A-C) or CD68 and VWF (D), signal was detected using the identical secondary antibody mixture (anti-mouse-IgG2a Alexa594, anti-rat-Alexa488 and anti-rabbit-Alexa647. All imaging capture and processing settings are identical. Panels A,A’ (rFVIII) and B,B’ (rFVIIIFc) show lack of FVIII signal from Kupffer cells (CD68, green), while negative controls in naïve mice C or dosed mice stained lacking primary anti-FVIII antibody (D) lack FVIII staining signal completely. rFVIII shows a vesicular staining in hepatocytes (A, A’), while rFVIIIFc shows a (patchy) sinusoidal staining pattern (B,B’). Merged images co-stained for Kuppfer cells (CD68) and VWF, show the complete lack of VWF signal, as expected in FVIII/VWF-DKO mice. For orientation: CV, central vein; HC, hepatocyte; KC, Kupffer cell; S, sinusoid. Scale bar, 20 μm. (TIF) [file pone.0124930.s006.tif]

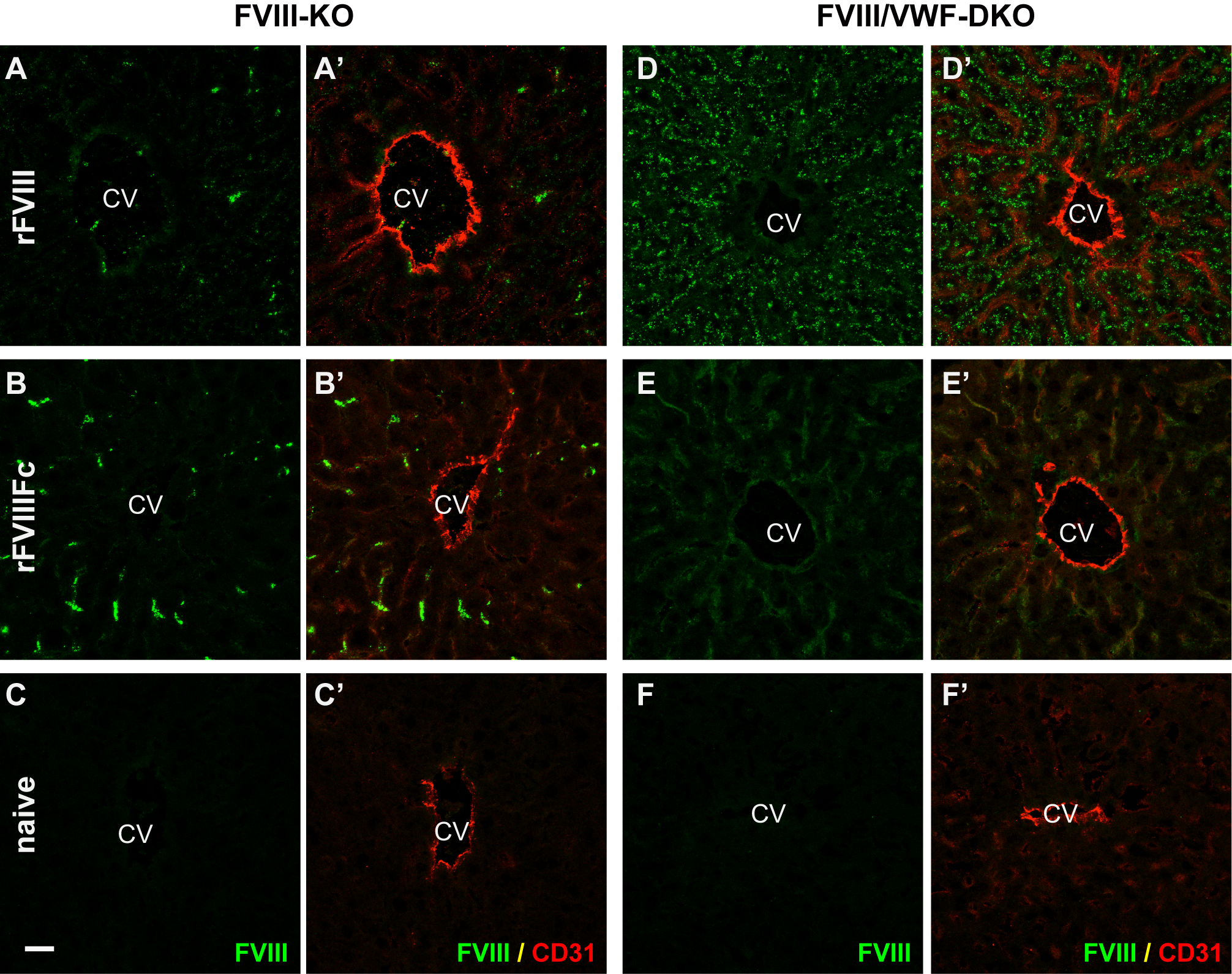

Supplement: S7 Fig — FVIII-KO (A-C) or FVIII/VWF-DKO mice (D-F) were dosed with equimolar amounts of rFVIII (296 μg/kg) (A, D) or rFVIIIFc (484 μg/kg) (B, E) or nothing (naïve, C, F). Five minutes post dosing mice were sacrificed and cryosections prepared, see material and methods. Sections were stained using identical staining conditions, primary antibody mixture against FVIII and CD31 signal was detected using the identical secondary antibody mixture (anti-mouse-IgG2a-Alexa488 and anti-rat-Alexa594. All imaging capture and processing settings are identical. In FVIII-KO mice (A-C), panels A and B show FVIII signal mostly in Kupffer cells, while no FVIII signal is detected in naïve mice C. A’-C’ are merged images for endothelial cell costaining (CD31). In FVIII/VWF-DKO mice (D-F) rFVIII signal is detected in hepatocytes (D, D’) and a fainter signal for rFVIIIFc is detected in sinusoids (E), costaining with endothelial cells (CD31, E’), no FVIII signal is detected in naïve sections of DKO mice (F, F’) For orientation: CV, central vein, scale bars, 20 μm. (TIF) [file pone.0124930.s007.tif]

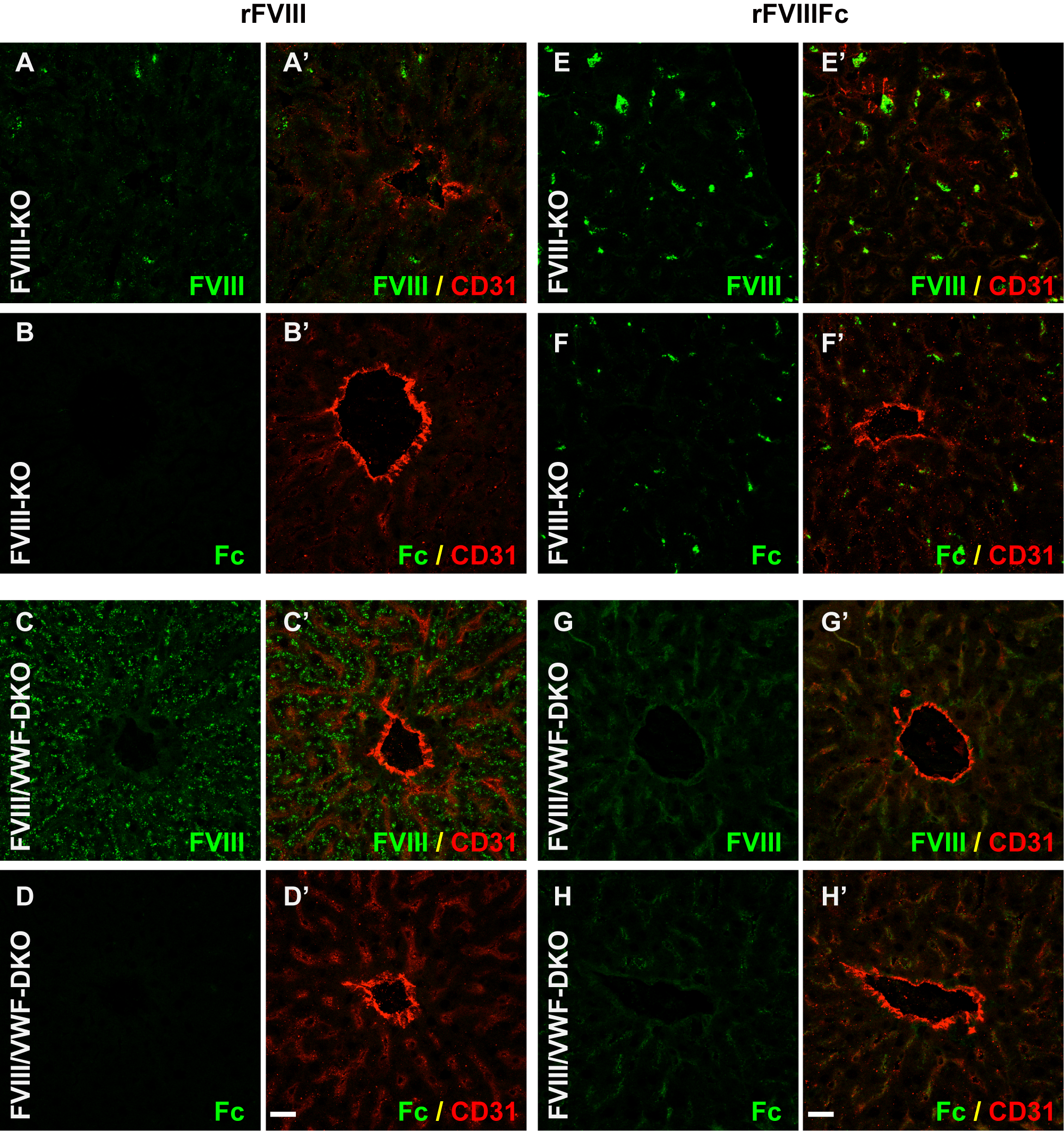

Supplement: S8 Fig — FVIII-KO (A-B, E-F) or FVIII/VWF-DKO mice (C-D, G-H) were dosed with equimolar amounts of rFVIII (296 μg/kg) (A-D) or rFVIIIFc (484 μg/kg) (E-H). Matched sections were stained using primary antibody mixture against FVIII and CD31 (A, C, E, G) or CD31 and human-IgG1 (B,D,F,H). In FVIII-KO mice gives anti-human Fc a Kupffer cell staining pattern for rFVIIIFc (F,F’) similar to that obtained using anti-FVIII (E). Negative control, is the lack of Kupffer cell signal in the anti-Fc staining (B) for rFVIII in as observed using anti-FVIII (A,A’). In FVIII/VWF-KO mice, sinusoidal staining of rFVIIIFc is detected by both anti-FVIII (G,G”) and Fc (H, H’). As a control, anti-Fc does not stain the strong hepatocyte vesicular pattern (D,D’) as observed for rFVIII in DKO mice (C,C’). Scale bars, 20 μm. (TIF) [file pone.0124930.s008.tif]

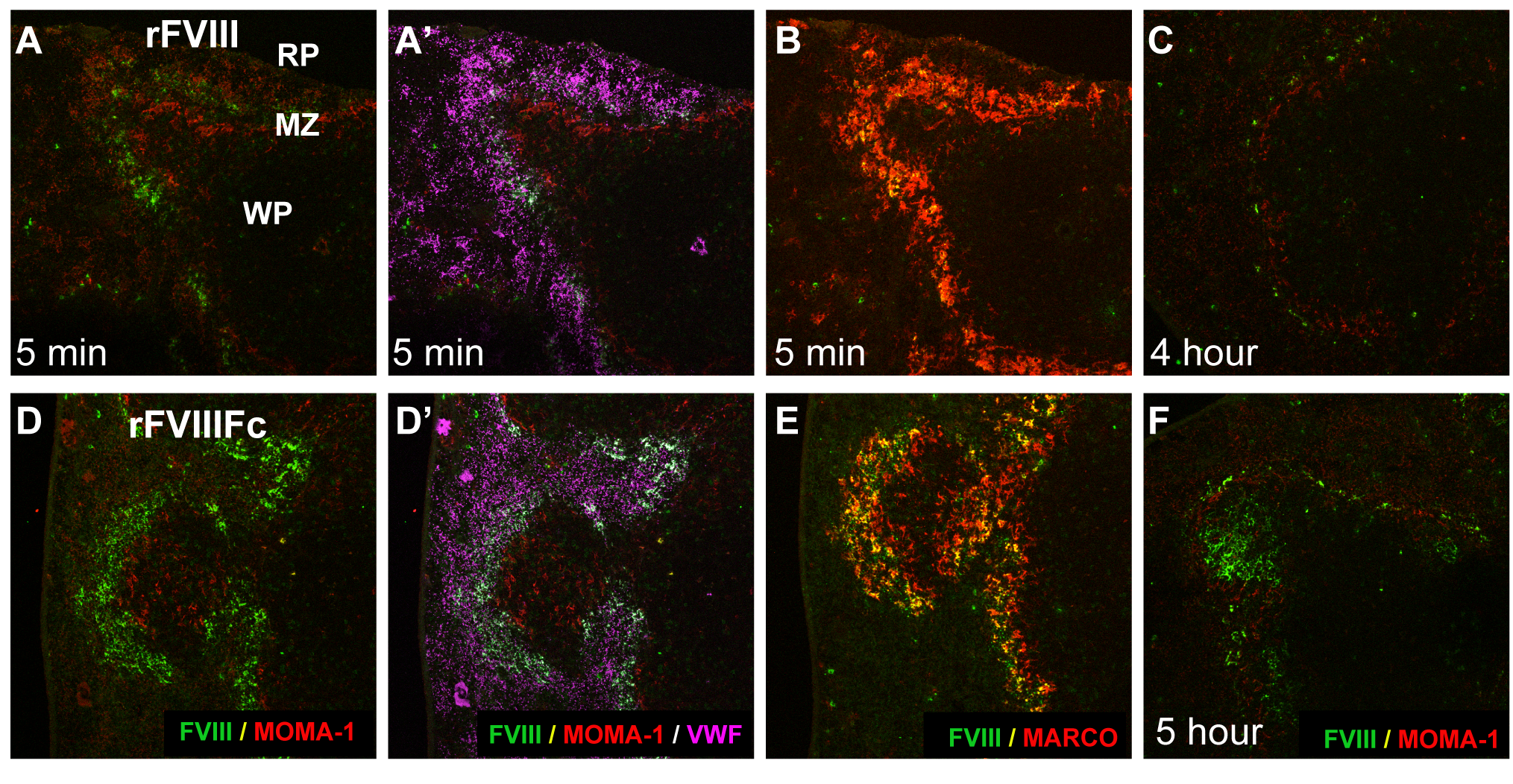

Supplement: S9 Fig — Both rFVIII (A-C) and rFVIIIFc (D-F) are predominantly internalized in MARCO+ (red) marginal zone (MZ) macrophages of the spleen (B, E) in FVIII-KO mice. VWF staining is absent in the metallophilic marginal zone macrophages stained with MOMA-1/CD169 (red) adjacent to the white pulp (WP) but instead extends from the FVIII and MARCO+ positive macrophages in the red pulp (RP) (A’,D’). rFVIII (C) and rFVIIIFc (F) staining (green) is still detected in a few marginal zone cells 4 and 5 hours after dosing. In FVIII/VWF-DKO mice neither rFVIII nor rFVIIIFc signal is detected in splenic macrophages at 5 minutes (not shown) similar to the VWF dependence of uptake by liver Kupffer cells. Original magnification is 200x. (TIF) [file pone.0124930.s009.tif]

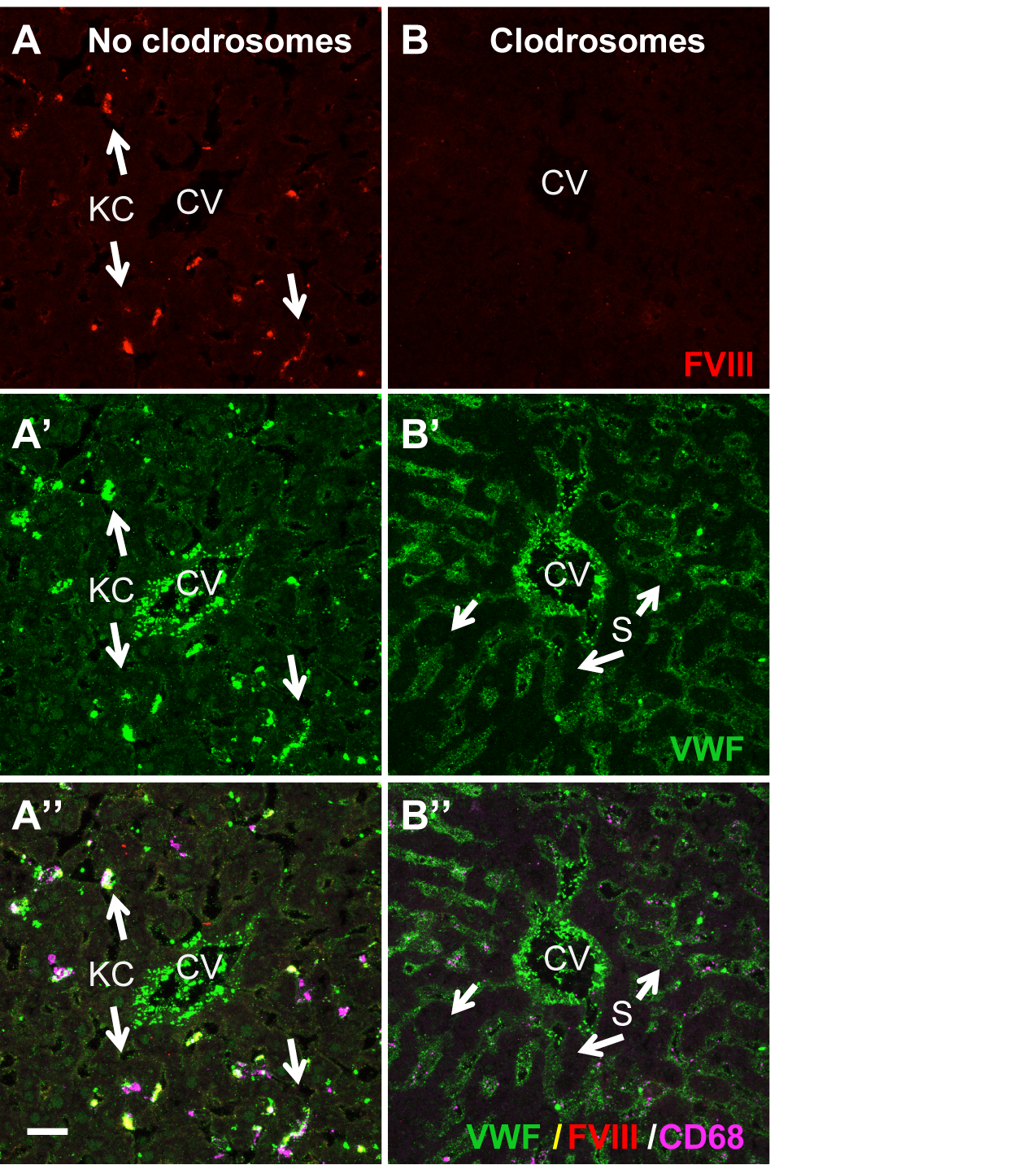

Supplement: S10 Fig — One day after clodrosome treatment, rFVIIIFc, endogenous VWF and Kupffer cells were stained in liver sections of FVIII-KO mice prepared 5 minutes after dosing rFVIIIFc. Control livers (A-A”) were compared to Kupffer cell depleted livers (B-B”). In control liver, rFVIIIFc is detected predominantly in Kupffer cells (A), along with endogenous VWF, which is also detected in the Weibel-Palade bodies of the large blood vessels (A’). An overlay of staining for rFVIIIFc, VWF and CD68, a Kuppfer cell marker, is shown (A”). In clodrosome treated FVIII-KO mice the rFVIIIFc signal is almost undetectable in liver (B) and endogenous VWF is no longer concentrated in Kupffer cells, as expected, but VWF staining remains in the endothelial cells of the larger vessels (B’). In addition, a relatively large increase in VWF staining is observed in liver sinusoid following clodrosome treatment. Decreased and diffuse staining of the Kupffer cell marker CD68 only on cell fragments confirms that clodrosome treatment depletes liver Kupffer cells (B”). For orientation in liver lobules: CV, central vein; S, sinusoid; KC and Kupffer cell (scale bar, 20 μm). (TIF) [file pone.0124930.s010.tif]
