# Supplementary material for: FcRn Rescues Recombinant Factor VIII Fc Fusion Protein from a VWF Independent FVIII Clearance Pathway in Mouse Hepatocytes
Source: PLoS One. 2015 Apr 23;10(4):e0124930. doi: 10.1371/journal.pone.0124930 (PMC4408089; doi:10.1371/journal.pone.0124930)
Supplement: S1 Material and Methods — (PDF) [file pone.0124930.s011.pdf]

FcRn Rescues Recombinant Factor VIII Fc Fusion Protein from a VWF Independent FVIII Clearance Pathway in Mouse Hepatocytes

Arjan van der Flier[1], Zhan Liu[1], Siyuan Tan[1], Kai Chen[1], Douglas Drager[1], Tongyao Liu[1], Susannah Patarroyo-White[1], Haiyan Jiang[1], David R. Light[1]

SUPPLEMENTAL MATERIAL AND METHODS

Clodrosome mediated Kupffer cell depletion

Kupffer cells and macrophages were depleted by a single intravenous dose of 10 mL/kg (50 mg/kg) clodrosomes, liposome encapsulated clodronate (Encapsula nanosciences LCC, Nashville, TN) and compared with control encapsomes, phosphate buffered saline loaded liposomes [1,2]. Mice were dosed 24 hours before the start of pharmacokinetic studies. Blood macrophage depletion was confirmed by flow cytometry of leucocytes following erythrocyte lysis. Tissue macrophage cell depletion was confirmed by qPCR analysis of RNA isolated from spleen, liver and lung. Kupffer cell depletion was quantified by immunohistochemical analysis of the surface area decrease in formalin liver sections stained for iba-1or F4/80 (not shown).

Supplemental References

1. Yamamoto T, Naito M, Moriyama H, Umezu H, Matsuo H, Kiwada H, et al. Repopulation of murine Kupffer cells after intravenous administration of liposome-encapsulated dichloromethylene diphosphonate. Am J Pathol. 1996 Oct;149(4):1271–86.

2. van Rooijen N, Hendrikx E. Liposomes for specific depletion of macrophages from organs and tissues. Methods Mol Biol. 2010;605:189–203.
