# Supplementary material for: FcRn Rescues Recombinant Factor VIII Fc Fusion Protein from a VWF Independent FVIII Clearance Pathway in Mouse Hepatocytes
Source: PLoS One. 2015 Apr 23;10(4):e0124930. doi: 10.1371/journal.pone.0124930 (PMC4408089; doi:10.1371/journal.pone.0124930)
Supplement: S1 Table — (PDF) [file pone.0124930.s012.pdf]

S1 Table. Antibodies used for immunohistochemistry and FACS

Murine antigen	Species	Clone	Vendor	Catalog number	Dilution
CD16/32	rat	2.4G2	BD Biosciences	553140	1:100, blocking
CD45.1/Ly5.1	mouse	A20	BD Biosciences	553775	1:100
CD45.2/Ly5.2	mouse	104	BD Biosciences	558702	1:100
CD90.1/Thy1.1	rat	OX-7	BD Biosciences	554897	1:100
CD90.2/Thy1.2	rat	535-2.1	BD Biosciences	553007	1:100
F4/80	rat	BM8	eBiosciences	53-4801/50-4801	1:20 (IHC)
F4/80	rat	CI:A3-1	AbD Serotec	MCA497A647, A488	1:100 (FACS)<br>1:10 (IHC)
CD68	rat	FA-11	AbD Serotec	MCA1957A488	1:10 with 2nd Ab
CD31	rat	ER-MP12	AbD Serotec	MCA2388A488	1:100 with 2nd Ab
CD31	rat	390	BD Biosciences	553708	1:50 with 2nd Ab
VWF	rabbit		Abcam	ab6994	1:400
Iba1	rabbit		Wako	019-19741	1:800 (paraformaldehyde)
human FVIII-A1	mouse		Green Mountain	GMA8004	1:400 with anti-IgG2
human FVIII-A2	mouse		Green Mountain	GMA8009	1:400
Human FVIII-C2	mouse		Green Mountain	GMA8018	1:400
Human FVIII-C2	mouse		Green Mountain	GMA8019	1:400
Moma-1/CD169	rat	Moma-1	AbD Serotec	MCA947	1:10
Marco	rat	ED31	AbD Serotec	MCA1849	1:100
mouse IgG2a	goat		Molecular Probes	A21131, A21135	1:200 (FVIII IHC)
rat-IgG	goat		Molecular Probes	A11006, A1107, A21247	1:200
rabbit-IgG	goat		Molecular Probes	A11013, A21244	1:200
human-IgG	goat		Molecular Probes	A11013	1:200
