# Supplementary material for: FcRn Rescues Recombinant Factor VIII Fc Fusion Protein from a VWF Independent FVIII Clearance Pathway in Mouse Hepatocytes
Source: PLoS One. 2015 Apr 23;10(4):e0124930. doi: 10.1371/journal.pone.0124930 (PMC4408089; doi:10.1371/journal.pone.0124930)
Supplement: S2 Table — (PDF) [file pone.0124930.s013.pdf]

S2 Table. LifeTechnologies qPCR primers for liver cell analysis

Murine Gene - Molecule	Probe Order Number
Fcgrt - IgG receptor FcRn large subunit	Mm00438887_m1
VWF - von Willibrand Factor	Mm00550376_m1
F8 – Factor VIII	Mm01215672_m1
LRP - Lrp1b	Mm00466712_m1
LDLR - Low density lipoprotein receptor	Mm00440169_m1
Asgr-1 - Asialoglycoprotein receptor	Mm01245581_m1
Stab2 - Stabilin-2	Mm00454684_m1
CD209a – DC-SIGN, DC-specific ICAM-3-grabbing non-integrin	Mm00460067_m1
Scara5 - Scavenger receptor-5	Mm00512272_m1
CLEC4F - C-type lectin domain family 4, member F	Mm00443934_m1
Siglec5 - Sialic acid binding Ig-like lectin 5	Mm00473131_m1
Alb - Albumin	Mm00802090_m1
LYVE-1 - Lymphatic vessel endothelial HA receptor-1	Mm00475056_m1
Emr-1, (F4/80) - Emerin homolog 1	Mm00802529_m1
CD68 - Macrosialin	Mm03047340_m1
GAPDH - Glyceraldehyde-3-phosphate dehydrogenase	Mm99999915_g1
B2m - β2 microglobulin	Mm00437762_m1
