# Supplementary material for: FcRn Rescues Recombinant Factor VIII Fc Fusion Protein from a VWF Independent FVIII Clearance Pathway in Mouse Hepatocytes
Source: PLoS One. 2015 Apr 23;10(4):e0124930. doi: 10.1371/journal.pone.0124930 (PMC4408089; doi:10.1371/journal.pone.0124930)
Supplement: S3 Table — (PDF) [file pone.0124930.s014.pdf]

S3 Table. Biodistribution of rFVIIIFc or rFVIII as calculated as %ID/organ by QWBA or scintillation counting FVIII-KO mice

FVIII-KO Mice	rFVIIIFc by QWBA (%ID/organ)					rFVIIIFc by Scintillation Counts (%ID/organ)			rFVIII by Scintillation Counts (%ID/organ)	
**Organ**	**5 min**	**30 min**	**3 hr**	**16 hr**	**32 hr**	**5 min**	**15 min**	**2 hr**	**15 min**	**1 hr**
**Blood**	**36.09**	**34.08**	**26.59**	**2.91**	**3.53**	**67.05 ±7.68**	**50.92 ±4.93**	**35.9 6±1.99**	**55.71 ±2.48**	**56.17 ±6.49**
**Liver**	**7.22**	**11.13**	**9.07**	**1.27**	**1.01**	**12.72 ±0.44**	**12.35 ±0.64**	**6.16 ±0.61**	**11.31 ±0.63**	**4.62 ±2.12**
Kidney	2.04	2.10	2.38	0.71	0.79	3.58 ±0.32	3. 0±0.11	2.38 ±0.37	2.85 ±0.11	2.57 ±0.03
Lung	1.09	1.48	1.18	0.16	0.10	0.54 ±0.23	0.42 ±0.03	0.32 ±0.02	0.23 ±0.05	0.41 ±0.38
Muscle	3.12	2.95	3.63	0.89	ND	5.48 ±1.24	4.43 ±0.4	5.76 ±1	4.11 ±0.4	2.99±0.26
Spleen	0.28	0.25	0.47	0.05	0.05	0.52 ±0.04	0.4 ±0.09	0.54 ±0.07	0.45 ±0.18	0.49 ±0.1
Heart	0.43	0.64	0.51	0.06	0.05	0.42 ±0.08	0.25 ±0.14	0.32 ±0.01	0.29 ±0.05	0.25 ±0.07
**Total (%ID)**	**50.27**	**52.63**	**43.83**	**6.05**	**5.53**	**90.28 ±10**	**71.87±6.35**	**51.53 ±4.09**	**75 ±3.9**	**67.54 ±9.45**
Urine						0.96±0.31	0.88±0.20	10	6.88±5.65	7.98±8.54
