# Supplementary material for: FcRn Rescues Recombinant Factor VIII Fc Fusion Protein from a VWF Independent FVIII Clearance Pathway in Mouse Hepatocytes
Source: PLoS One. 2015 Apr 23;10(4):e0124930. doi: 10.1371/journal.pone.0124930 (PMC4408089; doi:10.1371/journal.pone.0124930)
Supplement: S4 Table — (PDF) [file pone.0124930.s015.pdf]

S4 Table. Percentage of injected dose per gram of tissue (%ID/g) for rFVIIIFc or rFVIII as determined by QWBA or scintillation counting FVIII-KO mice

FVIII-KO Mice	rFVIIIFc by QWBA (%ID/g)					rFVIIIFc by Scintillation Counts (%ID/g)			rFVIII by Scintillation Counts (%ID/g)	
**Organ**	**5 min**	**30 min**	**3 hr**	**16 hr**	**32 hr**	**5 min**	**15 min**	**2 hr**	**15 min**	**1 hr**
**Blood**	**32.69**	**19.86**	**16.29**	**1.72**	**2.18**	**38.7±3.8**	**36.3±2.3**	**22.2±2.2**	**33.3±3.2**	**34.1±3.6**
**Liver**	**8.16**	**8.10**	**6.93**	**0.94**	**0.78**	**9.2±0.2**	**11±0.2**	**4.7±0.3**	**8.4±0.9**	**3.5±1.6**
Kidney	9.87	6.53	7.79	2.22	2.58	11±0.8	11.4±0.1	7.8±0	9.1±0.8	8.3±0
Lung	14.15	12.35	10.36	1.32	0.90	4.4±1.8	4.3±0.4	2.8±0.3	2.0±0.5	3.6±3.4
Muscle	0.57	0.34	0.44	0.1	ND	0.6±0.1	0.6±0.1	0.7±0	0.5±0	0.4±0
Spleen	4.83	2.76	5.47	0.56	0.55	5.7±0.5	5.4±1.5	6.3±0.1	5.1±1.8	5.7±1.2
Heart	7.3	6.95	05.87	0.63	0.56	4.5±0.9	3.2±1.7	3.7±0.7	3.2±0.7	2.8±0.8
Bile	2.86	5.16	13.50	0.63	0.84	ND	ND	ND	ND	ND
Cecum	0.23	0.34	9.97	0.42	0.31	ND	ND	ND	ND	ND
Large intestine	0.69	0.73	1.37	0.86	0.27	0.6±0.2	2.1±2.0	1.7±0	0.7±0	0.5±0.1
Urine	14.11	60.02	ND	1.14	3.75	146.5±33	72.5±41.2	167.4±	238.6±143.2	149.6±17.1
