# Supplementary material for: FcRn Rescues Recombinant Factor VIII Fc Fusion Protein from a VWF Independent FVIII Clearance Pathway in Mouse Hepatocytes
Source: PLoS One. 2015 Apr 23;10(4):e0124930. doi: 10.1371/journal.pone.0124930 (PMC4408089; doi:10.1371/journal.pone.0124930)
Supplement: S5 Table — (PDF) [file pone.0124930.s016.pdf]

S5 Table. Biodistribution of rFVIIIFc as calculated %ID/organ as determined by QWBA or scintillation counting in FVIII/VWF DKO Mice

**FVIII/VWF DKO mice**	**rFVIIIFc by QWBA (%ID/organ)**					**rFVIIIFc by Scintillation Counts (%ID/organ)**	
**Organ**	**5 min**	**15 min**	**1 hr**	**2 hr**	**6 hr**	**5 min**	**15 min**
**Blood**	**28.42**	**19.33**	**11.45**	**8.17**	**3.24**	**41.89±1.18**	**33.15±1.12**
**Liver**	**29.23**	**30.45**	**13.55**	**8.35**	**2.92**	**25.49±1.55**	**25.22±5.28**
Kidney	1.63	2.32	2.01	1.56	0.89	2.65±0.49	2.52±0.06
Lung	1.85	0.88	0.57	0.40	0.17	0.28±0.09	0.24±0.07
Muscle	3.59	2.57	1.73	2.57	1.57	3.91±0.77	3.4±0.21
Spleen	0.64	0.68	0.43	0.24	0.09	0.74±0.14	0.65±0.2
Heart	0.61	0.58	0.25	0.17	0.11	0.37±0.05	0.25±0.02
**Total (%ID)**	**65.97**	**56.81**	**29.99**	**21.46**	**8.99**	**75.5±4.32**	**65.46±7.01**
Urine						2.06±2.84	8.61±0.86
