# Supplementary material for: FcRn Rescues Recombinant Factor VIII Fc Fusion Protein from a VWF Independent FVIII Clearance Pathway in Mouse Hepatocytes
Source: PLoS One. 2015 Apr 23;10(4):e0124930. doi: 10.1371/journal.pone.0124930 (PMC4408089; doi:10.1371/journal.pone.0124930)
Supplement: S6 Table — (PDF) [file pone.0124930.s017.pdf]

S6 Table. Percentage of injected dose per gram of tissue (%ID/g) of rFVIIIFc as determined by QWBA or scintillation counting in FVIII/VWF DKO Mice

**FVIII/VWF DKO mice**	**rFVIIIFc by QWBA (%ID/g)**					**rFVIIIFc by Scintillation Counts (%ID/g)**	
**Organ**	**5 min**	**15 min**	**1 hr**	**2 hr**	**16 hr**	**5 min**	**15 min**
**Blood**	**22.98**	**9.18**	**5.91**	**4.04**	**1.51**	**19.9±0.9**	**16.5±0.7**
**Liver**	**29.50**	**18.04**	**8.73**	**5.16**	**1.70**	**15.1±0.7**	**15.7±3.4**
Kidney	7.02	5.87	5.55	4.11	2.22	6.7±1.4	6.7±0.1
Lung	21.35	5.94	4.18	2.83	1.13	1.9±0.6	1.7±0.5
Muscle	0.58	0.24	0.18	0.25	0.15	0.4±0.1	0.3±0
Spleen	9.93	6.14	4.20	2.24	0.81	6.7±1.4	6.2±1.9
Heart	7.30	6.95	5.87	0.63	0.56	3.3±0.5	2.3±0.2
Bile	5.48	3.56	18.06	18.99	14.37	ND	ND
Cecum	0.54	0.19	0.14	3.90	4.48	ND	ND
Large intestine	ND	0.52	0.30	1.43	8.77	0.4±0	0.4±0
Urine	55.24	50.20	3.55	99.22	4.84	43.3±53.8	168.4±123.9
